# σ-Bond electron delocalization of branched oligogermanes and germanium containing oligosilanes

**DOI:** 10.1016/j.ica.2014.07.005

**Published:** 2014-10-01

**Authors:** Johann Hlina, Rainer Zitz, Harald Wagner, Filippo Stella, Judith Baumgartner, Christoph Marschner

**Affiliations:** Institut für Anorganische Chemie, Technische Universität Graz, Stremayrgasse 9, A-8010 Graz, Austria; Institut für Chemie, Universität Graz, Stremayrgasse 9, A-8010 Graz, Austria

**Keywords:** Oligosilane, Oligogermane, Sigma-bond electron delocalization, UV-spectroscopy, X-ray crystal structures

## Abstract

In order to evaluate the influence of germanium atoms in oligo- and polysilanes, a number of oligosilane compounds were prepared where two or more silicon atoms were replaced by germanium. While it can be expected that the structural features of thus altered molecules do not change much, the more interesting question is, whether this modification would have a profound influence on the electronic structure, in particular on the property of σ-bond electron delocalization.

The UV-spectroscopic comparison of the oligosilanes with germanium enriched oligosilanes and also with oligogermanes showed a remarkable uniform picture. The expected bathochromic shift for oligogermanes and Ge-enriched oligosilanes was observed but its extent was very small. For the low energy absorption band the bathochromic shift from a hexasilane chain (256 nm) to a hexagermane chain with identical substituent patterns (259 nm) amounts to a mere 3 nm.

## Highlights

• Oligogermanes and germaoligosilanes isostructural to oligosilanes were synthesized.
• UV absorption data and X-ray diffraction revealed only marginal differences.
• Conformational flexibility is directly reflected in the UV absorption spectrum.

## Graphical abstract

A number of isostructural oligosilanes, germaoligosilanes, and oligogermanes were synthesized and studied by UV spectroscopy and X-ray diffraction.

**Figure:**
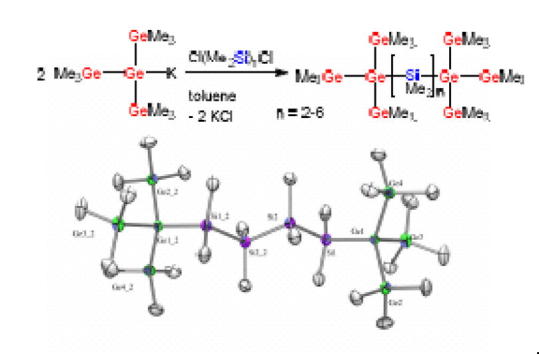

## 1 Introduction

Even a very superficial comparison of the organic chemistry of carbon and its higher congeners reveals immediately that common features are mostly restricted to the structural aspects of sp^3^-hybridized compounds. Although multiple bonds between higher group 14 elements are nowadays known it has to be emphasized that they do not match the ease of formation that is so characteristic for the carbon case [1]. In fact it has to be stated that organic molecules are more robust (i.e. have stronger element-element bonds), are more readily available, can easier be prepared and exhibit a much more delicate reactivity pattern.

However, there are some unique qualities of molecules containing bonds between higher group 14 elements. On the one hand they have a potential in organic synthesis being used as reagents, as protecting, masking, or directing groups [2]. On the other hand they have properties which can be attributed to the semiconducting nature of the parent elements. Oligo- and polymers containing connected chains of silicon, germanium, and tin atoms possess the unusual property of σ-bond electron delocalization. In a way related to the well known π-bond electron delocalization of organic conjugated molecules such as polyalkenes, this property is dependent on the conformation of the molecules [3]. This dependence has been studied theoretically by Michl and others [4], [5], [6]. In a series of elegant papers, Tsuji and Tamao prepared a number of conformationally constrained oligosilanes which unequivocally proved the concept of conformational dependence of σ-bond electron delocalization [7], [8], [9], [10], [11]. We and others have shown that oligosilanes with large end groups exhibit a preference to acquire a transoid conformation as long as the end groups are not too far apart from each other [6], [12], [13], [14].

An interesting question concerning the σ-bond electron delocalization is how much difference between oligosilanes, oligogermanes, and mixed compounds containing both silicon and germanium atoms can be expected. Related studies concerning the comparison of homo- and copolymers of silanes and germanes revealed a somewhat inconsistent picture [15], [16], [17]. However, the use of different substitution patterns and also different polymerization degrees complicates a reliable comparison. While recent progress in the synthesis of oligogermanes [18], [19], [20], [21], [22], [23], [24] has provided ready access to oligogermanes many of these contain chromophoric substituents [22], [23], [24], [25], [26], [27], complicating UV spectroscopic analysis. The use of isostructural oligomeric silanes, germanes, and germanium containing silanes should help to obtain a more accurate estimation of the influence of silicon and germanium on the nature of σ-bond electron delocalization. Utilizing synthetic methods developed in recent years for the preparation of small oligosilanes [28], [29], on several occasions we have synthesized compounds, which are structurally analogous to oligosilanes that have been studied before, but where one or several silicon atoms were being replaced by germanium atoms. Structural and spectroscopic characterization of these substances now allows their comparison with the respective all-silicon compounds and should thus reveal the influence of the added germanium atoms.

## 2 Results and discussion

### 2.1 Synthesis

**Scheme 1:**
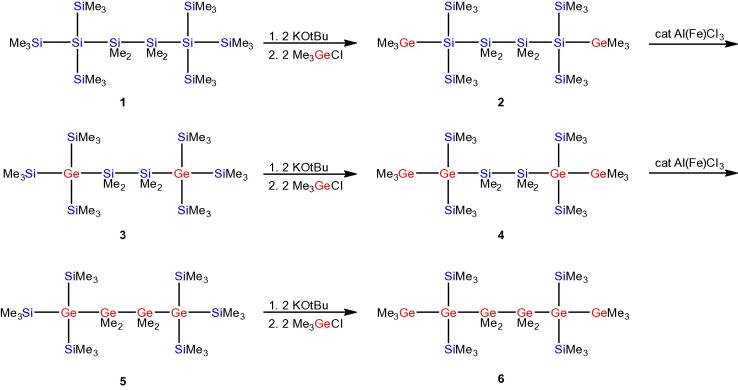
Synthesis of germanium containing structural analogs of 2,2,5,5,-tetrakis(trimethylsilyl)decamethylhexasilane.

In the course of our studies concerning the Lewis acid catalyzed rearrangement reactions of oligosilanes [30], [31], [32] we found that rearrangement of trimethylgermyl substituted oligosilanes led to the formation of silyl substituted germanes [33]. The deliberate introduction of germanium atoms was recognized as a unique way to generate germanium containing oligosilanes. Starting out from 2,2,5,5-tetrakis(trimethylsilyl)decamethylhexasilane (**1**), which represents the thermodynamic most stable oligosilane isomer of this composition, the replacement of two trimethylsilyl groups by trimethylgermyl groups gave the digermylated oligosilane **2** (Scheme 1). Lewis acid catalyzed rearrangement of **2** gave its isomer **3** with the germanium atoms at the quaternary positions. Replacing again two trimethylsilyl groups by trimethylgermyl groups gave compound **4** containing two digermanyl units. Rearrangement of **4** gave **5** with a central tetragermanylene unit (Scheme 1). Eventually replacement of two trimethylsilyl groups by trimethylgermyl groups concluded the synthesis of 2,2,5,5-tetrakis(trimethylsilyl)decamethylhexagermane (**6**) (Scheme 1) [33].

Compounds **1–6** are isostructural but contain different numbers of germanium atoms at different positions. Such a selection provides a formidable opportunity to study the influence of the replacement of silicon atoms by germanium atoms on the electronic properties by UV-spectroscopy. Crystal structure analysis of compounds **1** [34], [35], **3** and **5** [33] revealed that, not surprisingly, all compounds are isotypic. The distance between the two most highly separated silicon atoms of the *all-transoid* oriented chain segment may give an indication of how similar these compounds in fact are. The close similarity of these distances (**1**: 9.726 Å; **3**: 9.834 Å; **5**: 9.936 Å) already suggests a close resemblance of properties.

In addition to the rearrangement protocol described above, synthetic methodology of utilizing oligosilanyl and oligogermanyl anions [28] allowed the preparation of related compounds, where one or several silicon atoms are replaced by germanium. Again structural and in particular spectroscopic characterization allows comparison with the all-silicon compounds.

**Scheme 2:**
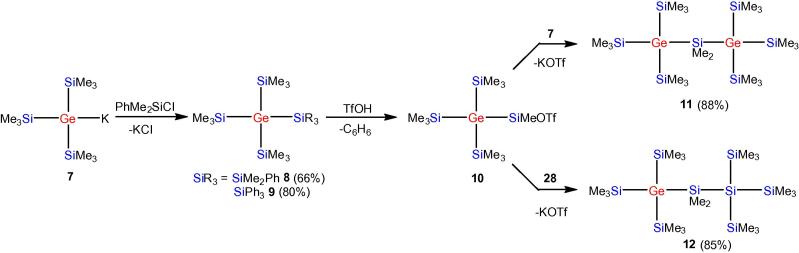
Synthesis of germanium containing structural analogs of 2,2,4,4,-tetrakis(trimethylsilyl)octamethylpentasilane.

**Scheme 3:**
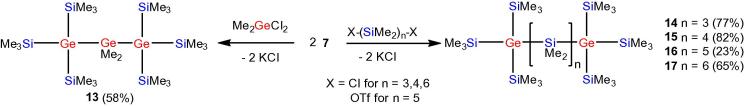
The use of tris(trimethylsilyl)germyl potassium **7** for the preparation of oligosilanylene connected bis[tris(trimethylsilyl)germyl] units.

Starting from easily available tris(trimethylsilyl)germyl potassium (**7**) [36] reaction with chlorodimethylphenylsilane and chlorotriphenyl silane gave dimethylphenylsilyltris(trimethylsilyl) germane (**8**) and triphenylsilyltris(trimethylsilyl)germane (**9**), respectively. Compound **8** was converted to the respective triflate **10** by reaction with trifluoromethanesulfonic acid [37] and further either with **7** or with tris(trimethylsilyl)silyl potassium (**28**) [38] to give the structurally related compounds **11** and **12** (Scheme 2). Reaction of two equivs of **7** with dichlorodimethylgermane gave another compound (**13**) of this series containing a trigermanylene unit at the core of the molecule (Scheme 3).

Reactions of two equivs of **7** with a series of linear α,ω-dichloropermethylsilanes [39] gave compounds where two tris(trimethylsilyl)germyl units are connected by oligosilanylene spacers of different length (Scheme 3) [12].

**Scheme 4:**
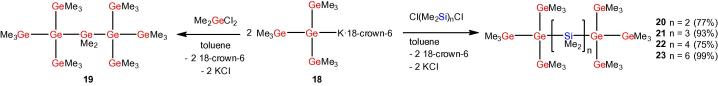
The use of tris(trimethylgermyl)germyl potassium **18** for the preparation of oligosilanylene connected bis[tris(trimethylgermyl)germyl] compounds.

Recently we extended the chemistry of branched oligosilanes to branched oligogermanes [40]. Utilizing two equivs of tris(trimethylgermyl)germyl potassium (**18**) as building blocks it was possible to prepare the branched oligogermane **19** by reaction with dichlorodimethylgermane (Scheme 4) [40]. Reaction of **18** with the same set of linear α,ω-dichloropermethylsilanes [39] as used above gave the oligosilanylene bridged compounds **20**–**23** (Scheme 4).

**Scheme 5:**
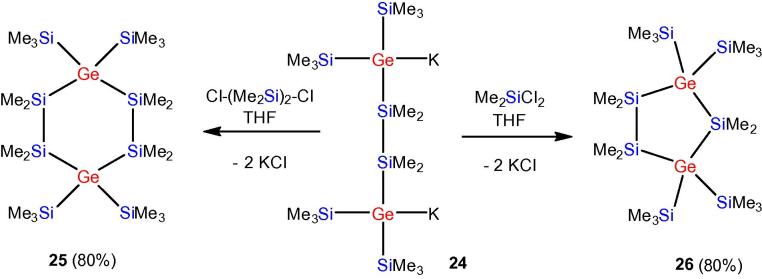
Synthesis of 1,4-digerma-1,1,4,4-tetrakis(trimethylsilyl)octamethylcyclohexasilane (**25**) and 1,3-digerma-1,1,3,3-tetrakis(trimethylsilyl)hexamethylcyclopentasilane (**26**).

The availability of oligosilanes with germanium atoms at branching points allows the facile formation of germyl dianions such as compound **24**. The latter is an excellent building block for the preparation of digermacyclosilanes. Analogous to the previously prepared homocyclohexa-[41] and -pentasilanes [42], [43] compounds **25** and **26** could be obtained by reaction with 1,2-dichlorotetramethyldisilane or dichlorodimethylsilane (Scheme 5).

**Scheme 6:**
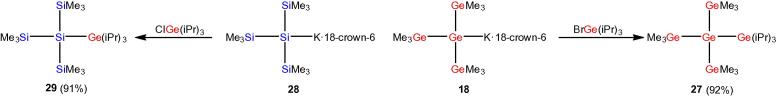
Preparation of tris(trimethylgermyl)triisopropylgermylgermane (**27**) and the structurally related tris(trimethylsilyl)triisopropylgermylsilane (**29**).

Using the described silyl and germyl anion chemistry it is also easy to prepare other oligosilane or oligogermane building blocks by reaction of the respective isotetrasilanyl or isotetragermanyl potassium compounds with trialkylgermyl halides such as chloro- or bromo triisopropylgermane. Compounds **27** and **29** were thus obtained in a facile way (Scheme 6).

### 2.2 UV–Vis spectroscopy

Although there are several reports concerned with UV–Vis absorption properties of oligogermanes [23], [24], [25], [26], [44], [45], [46], [47], [48], [49], [50], [51] it is difficult to make comparisons. Many of the studied compounds contain aryl substituents which interact with the σ-conjugated system [26]. However, comparison to peralkylated or permethylated systems [49], [50], [51] is possible.

**Fig. 1:**
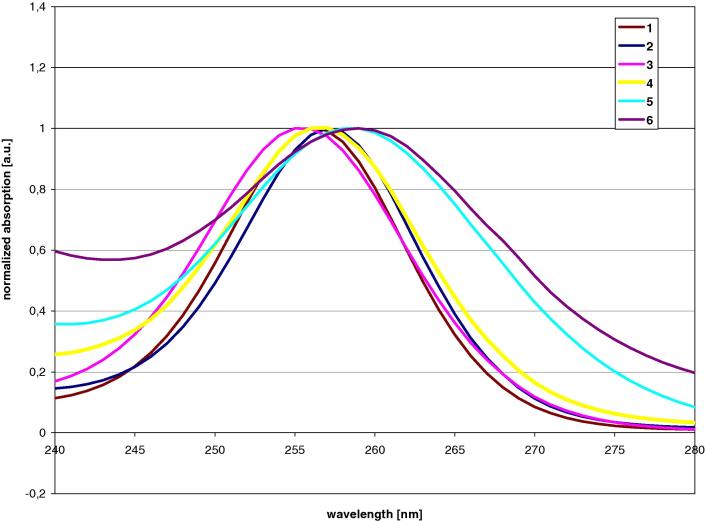
UV-spectra (selected section containing low energy absorption bands) of compound **1–6** with different numbers and positions of germanium atoms.

The assessment of the UV spectra of the isostructural compounds **1**–**6** revealed that the absorption bands corresponding to the σ-bond electron delocalization along the main chain are very similar (Fig. 1) covering a range from 255 to 259 nm (Table 1). While the hexagermane compound **6** exhibits the most bathochrome shifted absorption band (*λ* = 259 nm), compound **5** with the central tetragermanylene shows a more or less identical absorption wavelength. The absorption band with the lowest wavelength is not associated to the all-silicon compound **1** (*λ* = 256 nm) but to compound **3** (*λ* = 255 nm), where the germanium atoms are occupying the quaternary positions. The linear permethylated hexagermane Me(Me_2_Ge)_6_Me also exhibits its low energy band at 255 nm with a molar absorptivity of 2.5 × 10^4^ [51].

**Table 1:** Compilation of UV-absorption data.

Compound	Longest σ-delocalizing segment	Low energy absorption band (extinction [M^−1^ cm^−1^])	Other absorption bands (extinction [M^−1^ cm^−1^])
**1**	Si_6_	256 nm (8.2 × 10^4^)^a^	
**2**	Ge–Si_4_–Ge	257 nm (3.2 × 10^4^)^b^	
**3**	Si–Ge–Si_2_–Ge–Si	255 nm (1.1 × 10^5^)^b^	
**4**	Ge_2_–Si_2_–Ge_2_	256 nm (9.9 × 10^4^) b	
**5**	Si–Ge_4_–Si	256 nm (2.7 × 10^4^) b	
**6**	Ge_6_	259 nm (3.7 × 10^4^) b	
**11**	Si–Ge–Si–Ge–Si	245 nm (4.1 × 10^4^)	
**12**	Si–Ge–Si_3_	246 nm (3.4 × 10^4^)	
**13**	Si–Ge_3_–Si	253 nm (4.6 × 10^4^)	
**14**	Si–Ge–Si_3_–Ge–Si	269 nm (1.0 × 10^5^)	
**15**	Si–Ge–Si_4_–Ge–Si	279 nm (1.0 × 10^5^)	
**16**	Si–Ge–Si_5_–Ge–Si	286 nm (3.0 × 10^4^)	273 nm (3.0 × 10^4^)
**17**	Si–Ge–Si_6_–Ge–Si	294 nm (5.4 × 10^4^)	279 nm (4.4 × 10^4^)
**19**	Ge_5_	252 nm (4.7 × 10^4^) c	
**20**	Ge_2_–Si_2_–Ge_2_	259 nm (4.7 × 10^4^)	
**21**	Ge_2_–Si_3_–Ge_2_	272 nm (7.1 × 10^4^)	
**22**	Ge_2_–Si_4_–Ge_2_	282 nm (7.4 × 10^4^)	
**23**	Ge_2_–Si_6_–Ge_2_	296 nm (5.8 × 10^4^)	251 nm (2.5 × 10^4^), 282 nm (7.4 × 10^4^) shoulder)

a Values taken from Ref. [12].

b Values taken from Ref. [33].

c Values taken from Ref. [40].

**Fig. 2:**
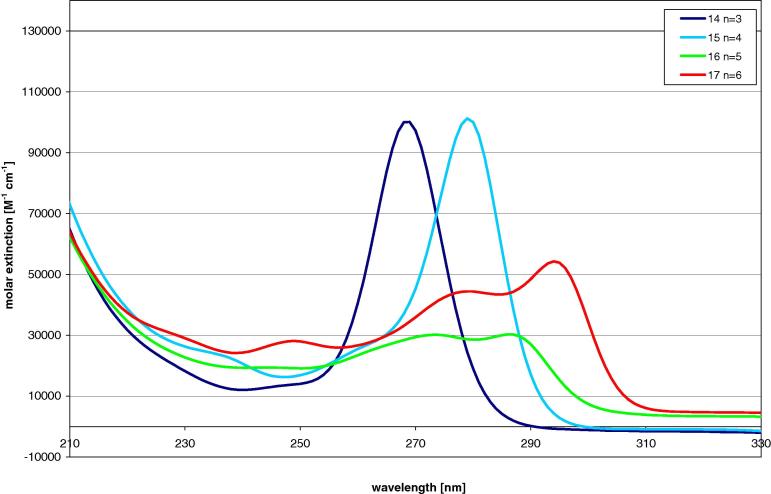
UV-spectra of compound **14**, **15**, **16**, and **17** with n dimethylsilylene spacer units.

UV spectroscopic investigation of oligosilanes consisting of tris(trimethylsilyl)silyl groups connected with one to six dimethylsilylene units showed that occurrence of only one bathochromic band associated with the existence of only one conformer for the molecules with up to four dimethylsilylene units (i.e. 1,4-tetrasilanylene spacer) (Fig. 2) (Table 1) [12], [13]. Longer spacers still cause a bathochromic shift of the lowest energy band consistent with a more extended σ-electron delocalized system. However, molecules with 1,5-pentasilanylene or 1,6-hexasilanylene spacers show in addition an absorption band associated with a conformer which does not feature an *all-transoid* conformation but rather corresponds to a conformation with a non *transoid-*aligned disilanylene unit (Fig. 2) [12]. The same behavior was observed previously for the oligosilanes with the same substitution patterns [12]. A comparison of the UV-spectroscopic properties of compounds **14**, **15**, **16**, and **17** to that of the all silicon compounds showed an almost complete congruence of the absorption traces (Fig. 4).

**Fig. 3:**
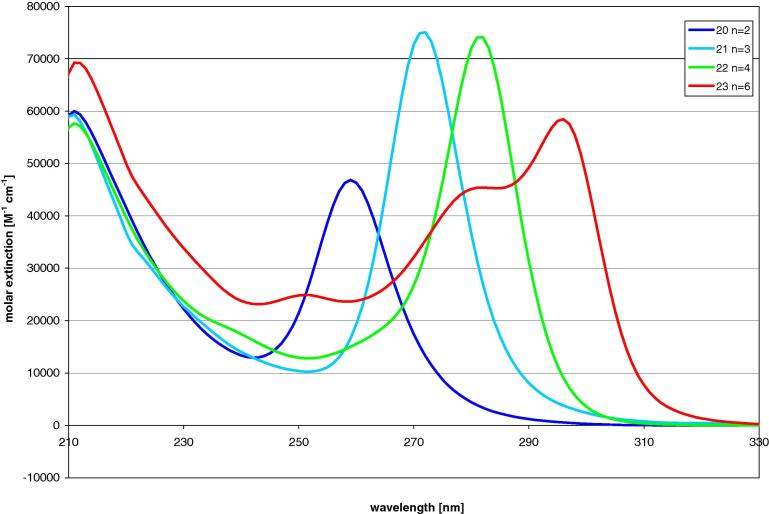
UV-spectra of compound **20**, **21**, **22**, and **23** with n dimethylsilylene spacer units.

**Fig. 4:**
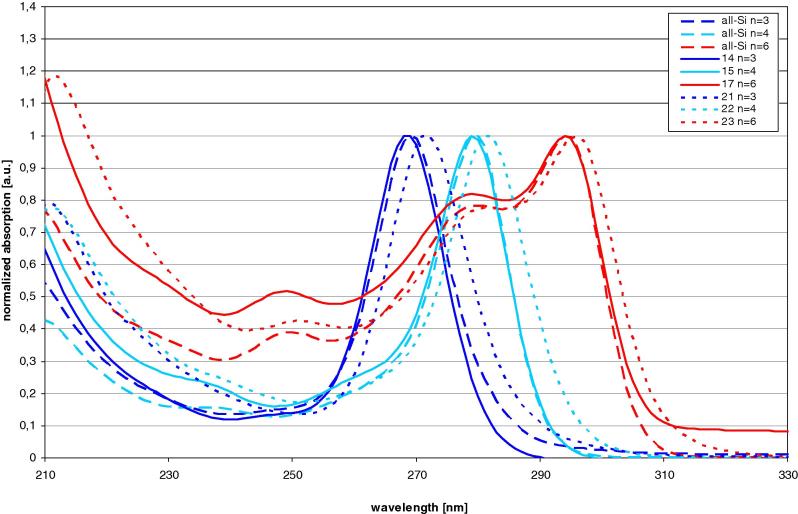
Comparison of UV-spectra of all-Si oligosilanes to compounds with tris(trimethylsilyl)germyl (**14**, **15**, **17**) and tris(trimethylgermyl)germyl groups (**21**, **22**, **23**).

The UV spectra of compounds **20**, **21**, **22**, and **23** (Fig. 3), which are different from compounds **14**–**17** as they contain tris(trimethylgermyl)germyl instead of tris(trimethylsilyl)germyl groups, look qualitatively very similar. Closer inspection reveals, however, a slight bathochromic shift of the low energy band (Fig. 4) (Table 1).

### 2.3 X-ray crystallography

Compounds **9**, **11**, **13**, **14**, **15**, **16**, **20**, **22**, **23**, **25**, **26**, **27**, and **29**, of this study were characterized by X-ray single-crystal structure analysis (see Table 2, Table 3). As numerous related polysilanes structures have been determined previously these compounds provide an excellent opportunity to compare structural properties of organooligosilanes and -germanes.

**Table 2:** Crystallographic data for compounds **9**, **11**, **13**, **14**, **15**, and **20**.

	**9**	**11**	**13**	**14**	**15**	**20**
Empirical formula	C_27_H_42_GeSi_4_	C_20_H_60_Ge_2_Si_7_	C_20_H_60_Ge_3_ Si_6_	C_24_H_72_Ge_2_Si_9_	C_26_H_78_Ge_2_Si_10_	C_22_H_66_Ge_8_Si_2_
*M*_w_	551.56	642.49	686.99	758.81	816.96	967.65
*T* (K)	100(2)	240(2)	100(2)	100(2)	100(2)	100(2)
Size (mm)	0.36 × 0.28 × 0.22	0.28 × 0.22 × 0.16	0.34 × 0.30 × 0.15	0.38 × 0.25 × 0.18	0.38 × 0.28 × 0.12	0.30 × 0.28 × 0.12
Crystal system	monoclinic	monoclinic	monoclinic	monoclinic	triclinic	monoclinic
Space group	*C*2/*c*	*C*2/*c*	*C*2/*c*	*C*2/*c*	$P\overset{¯}{1}$	*C*2/*c*
*a* (Å)	16.881(3)	17.055(3)	16.971(3)	15.585(3)	8.990(2)	16.022(3)
*b* (Å)	9.822(2)	9.285(2)	9.168(2)	9.899(2)	9.162(2)	9.915(2)
*c* (Å)	36.650(7)	24.665(5)	24.282(5)	58.11(2)	16.368(3)	26.387(5)
*α* (°)	90	90	90	90	82.10(3)	90
*β* (°)	91.48(3)	107.01(3)	106.44(3)	96.19(3)	75.60(3)	92.34(3)
*γ* (°)	90	90	90	90	66.72(3)	90
*V* (Å^3^)	6075(2)	3735(3)	3624(2)	8913(3)	1198(2)	4188(2)
*Z*	8	4	4	8	1	4
*ρ*_calc_ (g cm^−3^)	1.206	1.143	1.259	1.131	1.132	1.535
Absorption coefficient (mm^−1^)	1.180	1.842	2.679	1.604	1.519	5.726
F(000)	2336	1368	1440	3248	438	1928
*θ* Range	2.22 < *θ* < 26.37	1.735 < *θ* < 26.36	1.75 < *θ* < 26.35	0.70 < *θ* < 25.00	1.29 < *θ* < 26.36	2.42 < *θ* < 26.37
Reflections collected/unique	23713/6196	14488/3811	9481/3575	30826/7812	9565/4814	16071/4269
Completeness to *θ* [%]	99.7	99.8	96.8	100	98.3	99.5
Data/restraints/parameters	6196/0/298	3811/0/173	3575/0/167	7812/0/340	4814/0/185	4269/0/156
Goodness of fit (GOF) on F^2^	1.15	1.05	0.97	1.34	1.04	1.22
Final R indices [*I* > 2*σ*(*I*)]	*R*_1_ = 0.045, *wR*2 = 0.097	*R*_1_ = 0.033, *wR*_2_ = 0.0842	*R*_1_ = 0.057, *wR*_2_ = 0.111	*R*_1_ = 0.093, *wR*_2_ = 0.175	*R*_1_ = 0.030 *wR*_2_ = 0.071	*R*_1_ = 0.059 *wR*_2_ = 0.1089
R indices (all data)	*R*_1_ = 0.049, *wR*_2_ = 0.099	*R*_1_ = 0.039, *wR*_2_ = 0.087	R_1_ = 0.095, *wR*_2_ = 0.120	*R*_1_ = 0.104 *w*R2 = 0.179	*R*_1_ = 0.032 *wR*_2_ = 0.072	R_1_ = 0.076 *wR*_2_ = 0.112
Largest difference in peak/hole (e^−^/Å^3^)	0.67/−0.29	0.42/−0.17	0.91/−0.74	0.94/−1.37	0.68/−0.25	0.80/−0.73

**Table 3:** Crystallographic data for compounds **22**, **23**, **25**, **26**, **27**, and **29**.

	**22**	**23**	**25**	**26**	**27**	**29**
Empirical formula	C_26_H_78_Ge_8_Si_4_	C_30_H_90_Ge_8_Si_6_	C_30_H_90_Ge_3_Si_12_	C_18_H_54_Ge_2_Si_7_	C_18_H_48_Ge_5_	GeSi_4_C_18_H_48_
*M*_w_	1083.96	1200.28	1005.87	612.42	627.51	449.51
*T* (K)	150(2)	136(2)	293(2)	150(2)	100(2)	100(2)
Size (mm)	0.42 × 0.36 × 0.30	0.34 × 0.20 × 0.12	0.25 × 0.22 × 0.12	0.30 × 0.10 × 0.10	0.35 × 0.28 × 0.16	0.32 × 0.22 × 0.22
Crystal system	triclinic	monoclinic	triclinic	monoclinic	hexagonal	hexagonal
Space group	$P\overset{¯}{1}$	*P*2(1)/*c*	$P\overset{¯}{1}$	*P*2(1)/*c*	*R*3	*R*3
*a* (Å)	9.056(2)	14.566(3)	9.755(2)	9.228(2)	14.643(2)	14.513(2)
*b* (Å)	9.254(2)	9.724(2)	9.892(2)	32.901(7)	14.643(2)	14.513(2)
*c* (Å)	16.465(3)	41.632(8)	33.400(7)	12.868(5)	10.892(2)	10.772(3)
*α* (°)	82.49(3)	90	88.55(3)	90	90	90
*β* (°)	76.48(3)	94.27(3)	83.27(3)	117.97(2)	90	90
*γ* (°)	67.77(3)	90	66.97(3)	90	120	120
*V* (Å^3^)	1241(2)	5881(2)	2942(2)	3450(2)	2023(2)	1965(2)
*Z*	1	4	2	4	3	3
*ρ*_calc_ (g cm^−3^)	1.451	1.356	1.136	1.179	1.536	1.140
Absorption coefficient (mm^−1^)	4.887	4.170	1.785	1.991	5.509	1.352
F(000)	546	2440	1068	1296	948	732
*θ* Range	2.38 < *θ* < 26.29	1.40 < *θ* < 25.00	1.23 < *θ* < 26.36	1.90 < *θ* < 25.00	2.47 < *θ* < 26.29	2.49 < *θ* < 26.29
Reflections collected/unique	9813/4940	41131/10362	23717/11865	24511/6052	3950/1789	5024/1763
Completeness to *θ* [%]	98.3	99.9	98.6	100	100	100
Data/restraints/parameters	4940/0/185	10362/0/427	11865/0/543	6052/24/286	1789/1/76	1763/1/78
Goodness of fit (GOF) on F^2^	1.02	1.09	0.98	1.22	1.03	1.05
Final R indices [*I* > 2*σ*(*I*)]	*R*_1_ = 0.042, *wR*_2_ = 0.109	*R*_1_ = 0.096, *wR*_2_ = 0.144	*R*_1_ = 0.058, *wR*_2_ = 0.122	*R*_1_ = 0.116, *wR*_2_ = 0.236	*R*_1_ = 0.029, *wR*_2_ = 0.070	*R*_1_ = 0.023, *wR*_2_ = 0.058
R indices (all data)	*R*_1_ = 0.053, *wR*_2_ = 0.109	*R*_1_ = 0.127, *wR*_2_ = 0.170	*R*_1_ = 0.095, *wR*_2_ = 0.136	*R*_1_ = 0.130, *wR*_2_ = 0.244	*R*_1_ = 0.030 *wR*_2_ = 0.071	*R*_1_ = 0.024, *wR*_2_ = 0.058
Largest difference in peak/hole (e^−^/Å^3^)	1.53/−0.64	0.93/−0.56	0.71/−0.46	1.39/−1.08	0.71/−0.70	0.43/−0.19

**Fig. 5:**
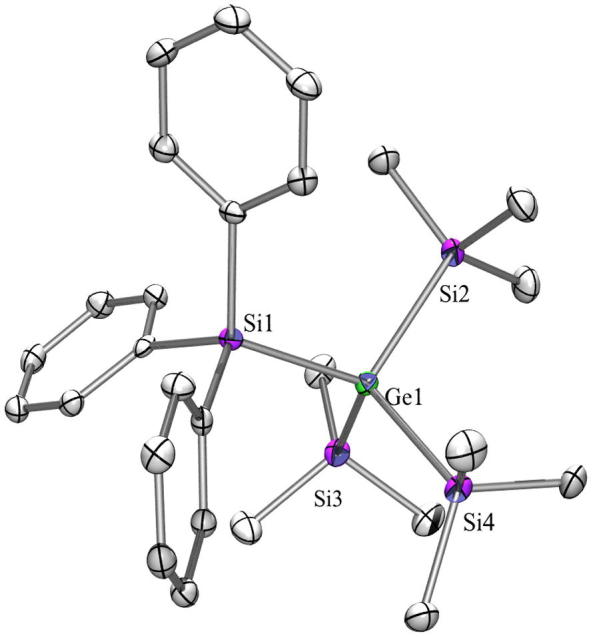
Crystal structure of **9**. Thermal ellipsoids are represented at the 30% level and hydrogen atoms have been omitted for clarity (bond lengths in Å, angles in deg). Ge(1)–Si(4) 2.3937(9), Ge(1)–Si(2) 2.3938(9), Ge(1)–Si(3) 2.3992(9), Ge(1)–Si(1) 2.4032(9), Si(1)–C(1) 1.884(3), Si(4)–Ge(1)–Si(2) 106.59(3), Si(4)–Ge(1)–Si(3) 108.65(3),Si(2)–Ge(1)–Si(3) 106.66(3), Si(4)–Ge(1)–Si(1) 113.15(3), Si(2)–Ge(1)–Si(1) 110.96(3), Si(3)–Ge(1)–Si(1) 110.55(4).

For the discussion of the structure of compound **9** (Fig. 5) it is interesting to note that the structure of tris(trimethylsilyl)triphenylsilylsilane [38], [52] has not been determined yet, while that of tris(trimethylsilyl)triphenylgermylsilane has [53]. The latter crystallized isotypically to **9** in the space group *C*2/*c*. The Ge–SiPh_3_ distance of 2.4031(9) Å found for **9** is close to the reported 2.416(1) Å for the Si-GePh_3_ bond [53].

**Fig. 6:**
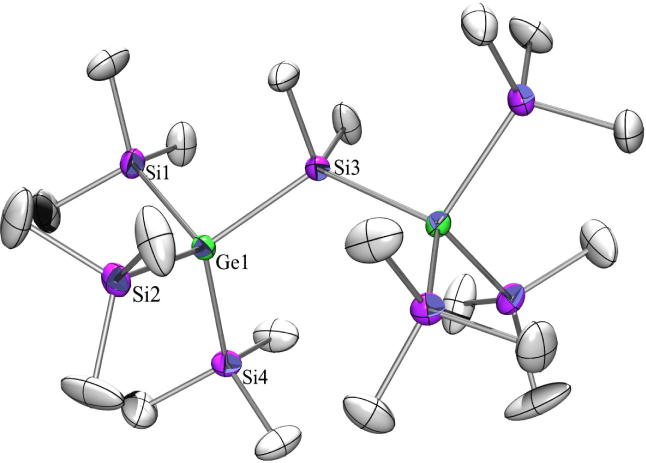
Crystal structure of **11**. Thermal ellipsoids are represented at the 30% level and hydrogen atoms have been omitted for clarity (bond lengths in Å, angles in deg). Ge(1)–Si(4) 2.3854(8), Ge(1)–Si(2) 2.4023(8), Ge(1)–Si(1) 2.4111(9), Ge(1)–Si(3) 2.4171(6), Si(1)–C(1) 1.860(3), Si(4)–Ge(1)–Si(2) 109.92(3), Si(4)–Ge(1)–Si(1) 105.49(4), Si(2)–Ge(1)–Si(1) 105.34(3), Si(4)–Ge(1)–Si(3) 115.54(3), Si(2)–Ge(1)–Si(3) 114.09(3), Si(1)–Ge(1)–Si(3) 105.44(3).

**Fig. 7:**
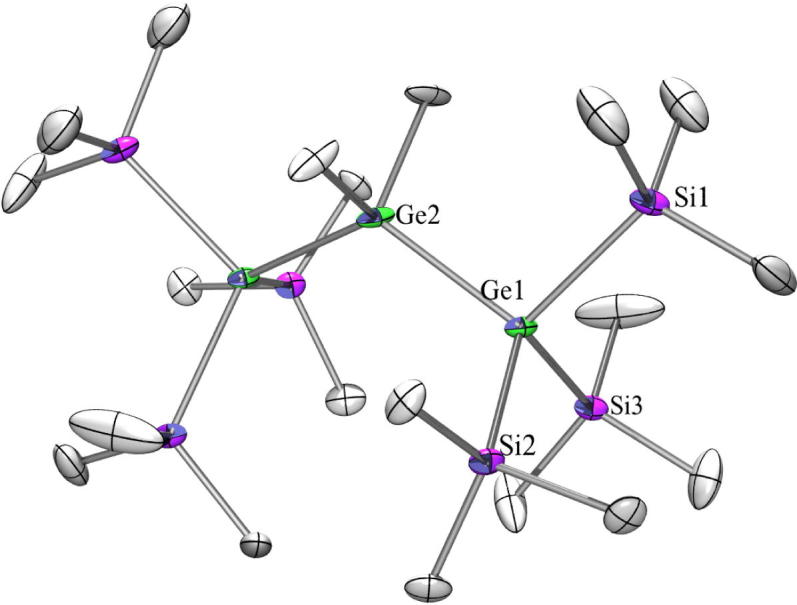
Crystal structure of **13**. Thermal ellipsoids are represented at the 30% level and hydrogen atoms have been omitted for clarity (bond lengths in Å, angles in deg).Ge(1)–Si(3) 2.3779(15), Ge(1)–Si(2) 2.3943(15), Ge(1)–Si(1) 2.4014(16), Ge(1)–Ge(2) 2.4616(8), Ge(2)–C(10) 1.979(6), Si(1)–C(2) 1.850(6), Si(3)–Ge(1)–Si(2) 111.07(5), Si(3)–Ge(1)–Si(1) 106.39(6), Si(2)–Ge(1)–Si(1) 106.36(6), Ge(1)–Ge(2)–Ge(1a) 125.00(4).

Compounds **11** (Fig. 6) and **13** (Fig. 7) as well as 1,1,1,3,3,3-hexakis(trimethylsilyl)-2,2-dimethyltrisilane [54] and the analogous all-germanium compound [40] crystallize all four in the monoclinic space group *C*2/*c* with half a molecule in the asymmetric unit in which one trimethylsilyl or trimethylgermyl group is disordered. The dihedral angles Me_3_Si-Ge-spacer-Ge(A)-Si(A)Me_3_ in **11** should be 60° in a perfect *gauche*-, 40° in a *cisoid*-, and 90° in an *ortho*-conformation [55]. For all four compounds a strong deviation from theses ideal values was found being 95.9°/32.8°, 95.9°/22.4°, and 90.0°/22.3° for **11** and 94.4°/33.8°, 95.4°/22.1°, and 91.3°/22.1° for **13.** For the two known compounds the angles are quite similar [40], [54]. The Ge–Si distances in **11** are between 2.39 Å and 2.41 Å for the Ge(SiMe_3_)_3_ group and with 2.42 Å for the Ge–SiMe_2_ distance the difference between ‘outer’ and ‘inner’ Ge–Si distances can be neglected.

The crystal structures of three different compounds with a Si_2_ or Ge_2_ spacer between the tris(trimethylsilyl)silyl or -germyl groups are reported in the literature to be triclinic ($P\overset{¯}{1}$) namely 1,1,1,4,4,4-hexakis(trimethylsilyl)-2,2,3,3-tetramethyltetrasilane [34], [35], 1,2-bis[tris(trimethylsilyl)germyl]tetramethyldisilane [33], and 1,2-bis[tris(trimethylsilyl)germyl]tetramethyldigermane [33]. In contrast to these three compounds **20** (Fig. 10) with a Si_2_ spacer between the two isotetragermyl groups is crystallizing in the monoclinic space group *C*2/*c* with half a molecule in the asymmetric unit. The dihedral angles Me_3_*Ge*-*Ge*-[SiMe_2_]_2_-*Ge*-*Ge*Me_3_ in **20** exhibit a deviation from the 60° of a perfectly staggered conformation (62.8°, 66.0°, 51.2°) and are close to the values found for the corresponding other three compounds.

For all structures in this manuscript bearing a Ge–Ge bond the distances are all with about 2.44 Å in the range published for a couple of branched oligogermanes [40] and perphenylated linear and branched oligogermanes [24], [56].

**Fig. 8:**
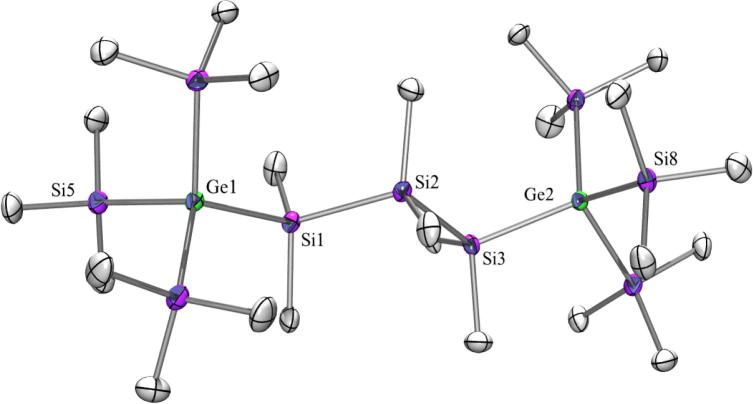
Crystal structure of **14**. Thermal ellipsoids are represented at the 30% level and hydrogen atoms have been omitted for clarity (bond lengths in Å, angles in deg). Ge(1)–Si(5) 2.393(2), Ge(1)–Si(1) 2.394(2), Ge(2)–Si(8) 2.379(2), Ge(2)–Si(3) 2.392(2), Si(1)–C(2) 1.886(8), Si(1)–Si(2) 2.356(3), Si(2)–Si(3) 2.370(3), Si(5)–Ge(1)–Si(1) 106.14(8), Si(8)–Ge(2)–Si(3) 114.45(8), Si(2)–Si(1)–Ge(1) 119.44(10), Si(1)–Si(2)–Si(3) 106.22(11), Si(2)–Si(3)–Ge(2) 118.20(10).

Compound **14** (Fig. 8) with a Si_3_ spacer between the tris(trimethylsilyl)germyl groups and the corresponding all Si compound [54] are both crystallizing in the monoclinic space group *C*2/*c*. Unfortunately, no structure could be obtained from compound **21**. The dihedral angles for the all silicon compound Me_3_Si–Si⋯Si–SiMe_3_ were reported to be all between 56.6° and 66.0° [54] and in **14** they are between 66.5° and 57.1° and show a staggered conformation with respect to the tris(trimethylsilyl)silyl and tris(trimethylsilyl)germyl groups, respectively.

**Fig. 9:**
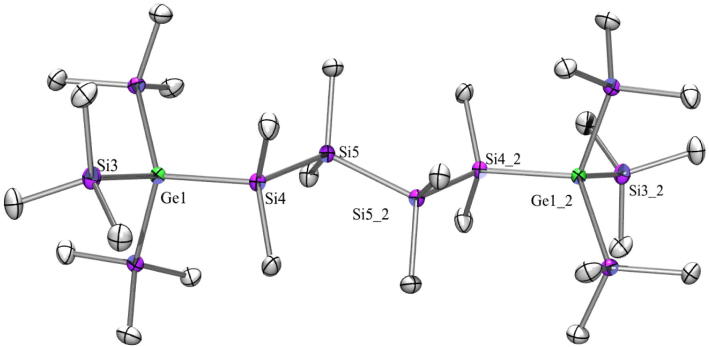
Crystal structure of **15**. Thermal ellipsoids are represented at the 30% level and hydrogen atoms have been omitted for clarity (bond lengths in Å, angles in deg). Ge(1)-Si(3) 2.3905(8), Ge(1)–Si(4) 2.3925(12), Si(1)–C(8) 1.874(2), Si(4)–Si(5) 2.3620(10), Si(5)–Si(5_2) 2.3539(15), Si(3)–Ge(1)–Si(4) 107.45(3), Si(5)–Si(4)–Ge(1) 116.26(3), Si(5_2)–Si(5)–Si(4) 109.06(5).

The next molecules in this series are **15** (Fig. 9) and **22** (Fig. 11) with four dimethylsilylene units as spacer. Both as well as the corresponding all silicon compound [54] crystallize in the triclinic space group $P\overset{¯}{1}$. For all three the asymmetric unit consists of half a molecule with an inversion center in the middle of the central spacer chain bond.

**Fig. 10:**
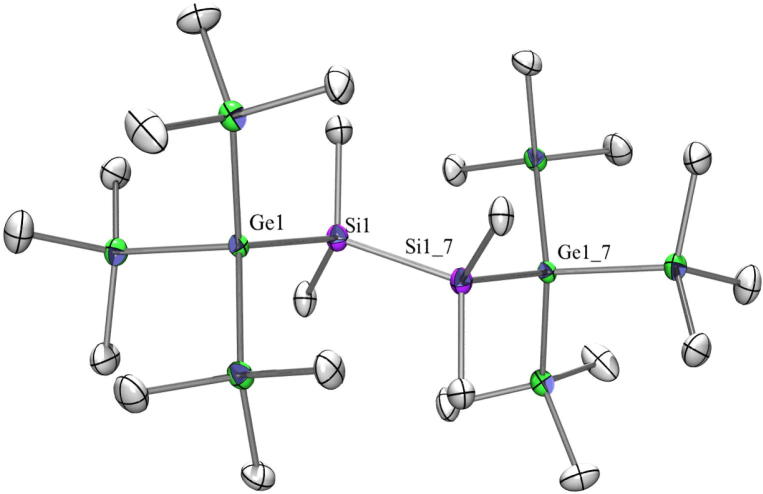
Crystal structure of **20**. Thermal ellipsoids are represented at the 30% level and hydrogen atoms have been omitted for clarity (bond lengths in Å, angles in deg). Ge(1)–Si(1) 2.4103(19), Ge(1)–Ge(2) 2.4320(10), Ge(1)–Ge(3) 2.4369(11), Ge(1)–Ge(4) 2.4434(10), Ge(2)–C(1) 1.959(7), Si(1)–Si(1_7) 2.349(3), Si(1)–Ge(1)–Ge(2) 116.25(5), Si(1_7)–Si(1)–Ge(1) 114.81(11).

**Fig. 11:**
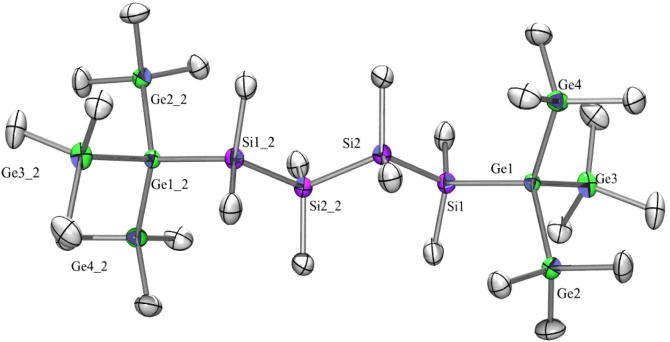
Crystal structure of **22**. Thermal ellipsoids are represented at the 30% level and hydrogen atoms have been omitted for clarity (bond lengths in Å, angles in deg). Ge(1)–Si(1) 2.4053(16), Ge(1)–Ge(4) 2.4370(11), Ge(1)–Ge(2) 2.4374(10), Ge(1)–Ge(3) 2.4435(8), Ge(2)–C(1) 1.949(4), Si(1)–C(10) 1.879(5), Si(2)–Si(2_2) 2.346(3), Si(1)–Ge(1)–Ge(4) 115.56(4), Si(1)–Ge(1)–Ge(2) 110.98(4), Ge(4)–Ge(1)–Ge(2) 110.52(3), Si(1)–Ge(1)–Ge(3) 107.26(4), Ge(4)–Ge(1)–Ge(3) 106.59(3), Ge(2)–Ge(1)–Ge(3) 105.26(3), Si(2)–Si(1)–Ge(1) 114.60(6), Si(2_2)–Si(2)–Si(1) 109.49(8).

**Fig. 12:**
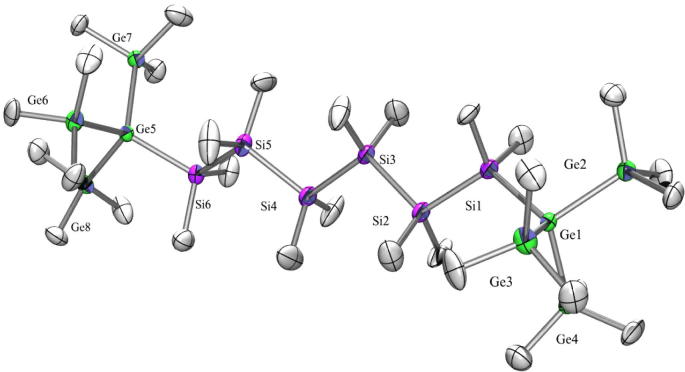
Crystal structure of **23**. Thermal ellipsoids are represented at the 30% level and hydrogen atoms have been omitted for clarity (bond lengths in Å, angles in deg). Ge(1)–Si(1) 2.404(4), Ge(1)–Ge(3) 2.4278(18), Ge(1)–Ge(4) 2.4319(17), Ge(1)–Ge(2) 2.4417(17), Ge(2)–C(1) 1.940(11), Ge(5)–Si(6) 2.397(3), Ge(5)–Ge(7) 2.4336(18), Ge(5)–Ge(6) 2.4341(17), Ge(5)–Ge(8) 2.4424(17), Si(1)–Si(2) 2.349(5), Si(2)–Si(3) 2.345(5), Si(3)–Si(4) 2.348(5), Si(4)–Si(5) 2.339(5), Si(5)–Si(6) 2.342(5), Si(1)–Ge(1)–Ge(3) 112.90(10), Si(1)–Ge(1)–Ge(4) 115.29(10), Ge(3)–Ge(1)–Ge(4) 108.64(7), Si(1)–Ge(1)–Ge(2) 107.28(10), Ge(3)–Ge(1)–Ge(2) 105.04(6), Ge(4)–Ge(1)–Ge(2) 107.02(6), Si(6)–Ge(5)–Ge(6) 116.94(10), Ge(7)–Ge(5)–Ge(6) 108.62(6), Si(6)–Ge(5)–Ge(8) 106.05(9), Ge(6)–Ge(5)–Ge(8) 104.49(6), Si(2)–Si(1)–Ge(1) 116.20(16), Si(3)–Si(2)–Si(1) 110.84(18), Si(2)–Si(3)–Si(4) 110.81(18), Si(5)–Si(4)–Si(3) 108.97(18), Si(4)–Si(5)–Si(6) 110.72(18), Si(5)–Si(6)–Ge(5) 116.55(16).

As the compounds dealt with in this study are rather non-polar, with longer chain lengths they are also less soluble. This is known from polysilanes and usually does not facilitate crystallization. Compound **23** (Fig. 12) with a hexasilanylene spacer between the tris(trimethylsilyl)germyl groups crystallized in the monoclinic space group *P*2(1)/*c*, which is in contrast to the corresponding all-silicon compound which crystallized in the space group P1 [13]. Both exhibit a regular all-transoid arrangement of the spacer segments and a nearly perfect *ortho*-conformation of the tris(trimethylsilyl)germyl or tris(trimethylsilyl)silyl groups.

**Fig. 13:**
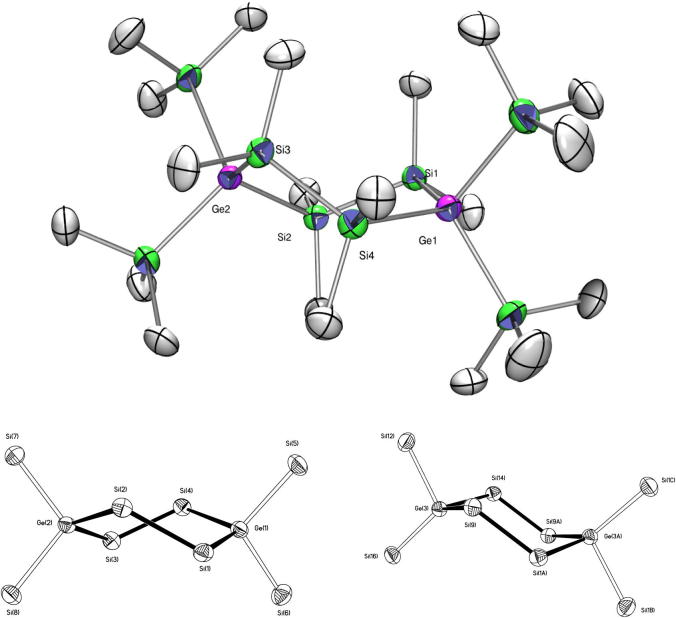
Crystal structure of **25**. Thermal ellipsoids are represented at the 30% level and hydrogen atoms have been omitted for clarity (bond lengths in Å, angles in deg). Ge(2)–Si(2) 2.3845(14), Ge(2)–Si(3) 2.3888(15), Ge(2)–Si(8) 2.3902(14), Ge(1)–Si(6) 2.3896(16), Ge(1)–Si(1) 2.3937(16), Ge(1)–Si(4) 2.3944(15), Si(1)–C(1) 1.890(4), Si(1)–Si(2) 2.3398(18), Si(3)–Si(4) 2.3436(19), Si(2)–Ge(2)–Si(3) 111.41(5), Si(8)–Ge(2)–Si(7) 106.16(6), Si(5)–Ge(1)–Si(6) 106.39(6), Si(6)–Ge(1)–Si(4) 110.94(5), Si(1)–Ge(1)–Si(4) 111.73(6), Si(2)–Si(1)–Ge(1) 112.80(6), Si(1)–Si(2)–Ge(2) 114.00(6), Si(4)–Si(3)–Ge(2) 113.40(6), Si(3)–Si(4)–Ge(1) 113.52(6).

Exchanging a trimethylsilyl group in tetrakis(trimethylsilyl)silane by a triisopropylsilyl group lowers the symmetry from cubic to trigonal (R3) [54]. Tris(trimethylgermyl)triisopropylsilylgermane [40], **27** (Fig. 15) and **29** (Fig. 16) also crystallize in the same space group (trigonal, R3) with nearly the same cell dimensions. The bond angles are 106.4° for Me_3_Si–Si–SiMe_3_ in **29**, 105.7° for Me_3_Ge–Ge–GeMe_3_ in **27**, 112.4° for Me_3_Si–Si–Ge*^i^*Pr_3_ in **29**, and 113.0° for Me_3_Ge–Ge–Ge*^i^*Pr_3_ in **27** and thus practically identically with the ones in tris(trimethylgermyl)triisopropylsilylgermane [40] and in tris(trimethylsilyl)triisopropylsilylsilane [54].

**Fig. 14:**
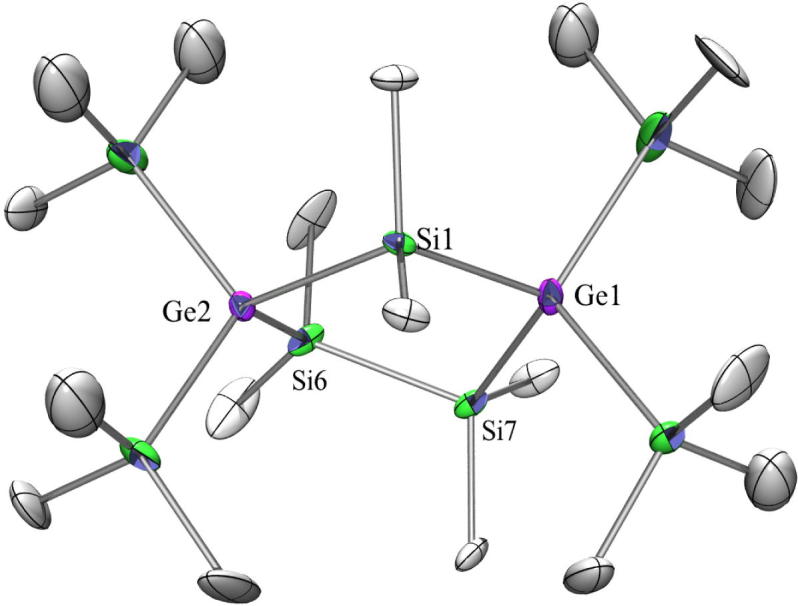
Crystal structure of **26**. Thermal ellipsoids are represented at the 30% level and hydrogen atoms have been omitted for clarity (bond lengths in Å, angles in deg). Si(6)–C(16) 1.737(16), Si(6)-Si(7) 2.328(10), Si(6)–Ge(2) 2.342(7), Si(7)–Ge(1) 2.497(7), Ge(1)–Si(1) 2.387(3), Ge(2)–Si(1) 2.390(3), Si(7)–Si(6)–Ge(2) 107.0(3), Si(6)–Si(7)–Ge(1) 102.7(3), Si(2)–Ge(1)–Si(7) 102.0(2), Si(1)–Ge(1)–Si(7) 104.77(17), Si(6)–Ge(2)–Si(1) 105.09(18), Ge(1)–Si(1)–Ge(2) 107.52(11).

**Fig. 15:**
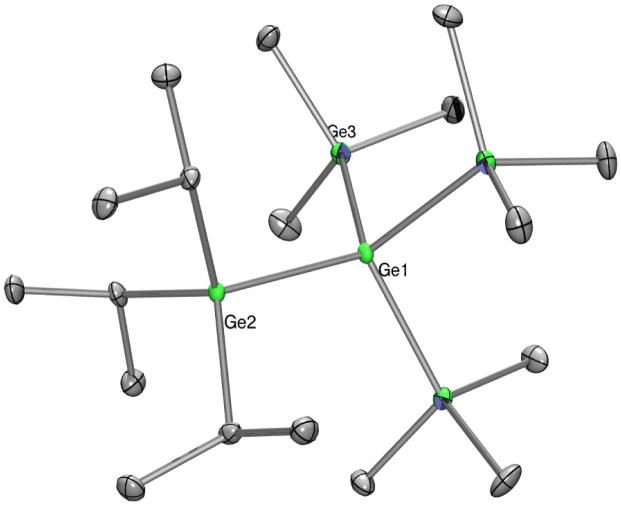
Crystal structure of **27**. Thermal ellipsoids are represented at the 30% level and hydrogen atoms have been omitted for clarity (bond lengths in Å, angles in deg). Ge(1)–Ge(3) 2.4334(5), Ge(1)–Ge(2) 2.4442(10), Ge(2)–C(1) 1.989(4), Ge(3)–Ge(1)–Ge(2) 113.043(16), Ge(3)–Ge(1)–Ge(3_1) 105.674(18).

**Fig. 16:**
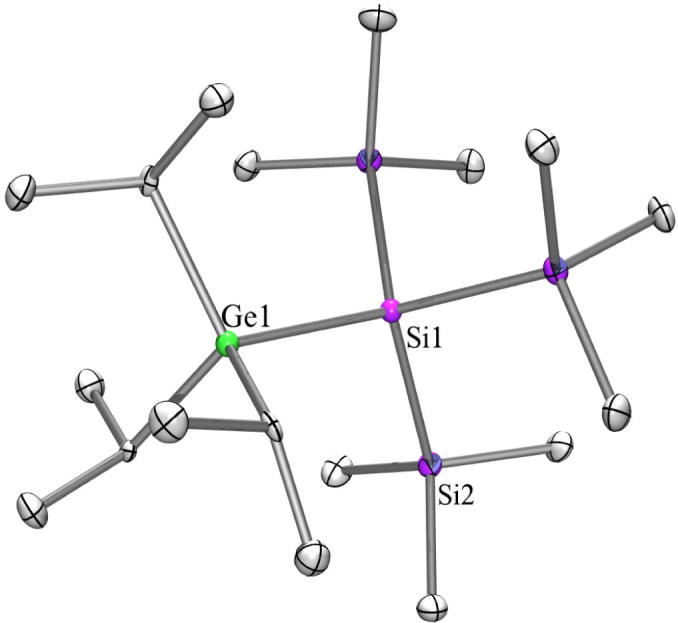
Crystal structure of **29**. Thermal ellipsoids are represented at the 30% level and hydrogen atoms have been omitted for clarity (bond lengths in Å, angles in deg). Ge(1)–C(4) 2.035(2), Ge(1)–Si(1) 2.4148(12), Si(2)–C(1) 1.878(3), Si(2)–Si(1) 2.3621(8), Si(2)–Si(1)–Ge(1) 112.38(3), Si(2_1)–Si(1)–Si(2) 106.41(3).

The five-membered ring **26** (Fig. 14) with two quaternary germanium atoms crystallized in the monoclinic space group *P*2(1)/*c* whereas the analogous all silicon five-membered ring and 1-germa-2,2,4,4-tetrakis(trimethylsilyl)hexamethylcyclopentasilane are both reported to crystallize in *C*2/*c* [57]. Unfortunately, the obtained data are of low quality and the –Me_2_Si–Me_2_Si– part of the ring is disordered causing some restraints in the structure solution. The ring engaged in an envelope conformation with on of the disordered Me_2_Si group on the flap.

The typical conformation for 1,4-substituted cyclohexasilanes is a chair with the large substituents in equatorial positions. 1,1,4,4,-Tetrakis(trimethylsilyl)octamethylcyclohexasilane, which crystallized in the space group *C*2/*c* is an exception. As all four trimethylsilyl groups have the same steric demand the system adopts a twist conformation [58]. For compound **25** (Fig. 13) crystallizing in $P\overset{¯}{1}$ the situation is somewhat unusual. With one and a half molecule in the asymmetric unit one of these molecules adopts a twist while the other one prefers a chair conformation (Fig. 13).

## 3 Conclusion

In recent years a number of fundamental studies have provided a much better understanding of the property of σ-bond electron delocalization in polysilanes [3], [4], [5], [7], [8], [9], [10], [11]. While the situation for polygermanes and polystannanes is certainly very similar to polysilanes there are also differences to expect. On the one hand the longer Ge–Ge and in particular Sn–Sn bonds allow for a different conformational behavior and on the other hand the different energy levels of higher orbitals will certainly have an impact.

The current study was devoted to an investigation of the influence of the replacement of silicon atoms in oligosilanes by germanium atoms. As expected the structural features of molecules altered this way were almost identical. The more interesting question of the influence of this modification on the electronic structure and the property of σ-bond electron delocalization was analyzed using UV-spectroscopy. Comparison of the UV spectra of isostructural oligosilanes with germanium enriched oligosilanes and with silyl substituted oligogermanes showed that they were almost identical with the expected but almost negligible bathochromic shift of absorption bands for germanium enriched compounds.

## 4 Experimental

### 4.1 General remarks

All reactions involving air-sensitive compounds were carried out under an atmosphere of dry nitrogen or argon using either Schlenk techniques or a glove box. Solvents were dried using a column solvent purification system [59].

^1^H (300 MHz), ^13^C (75.4 MHz), and ^29^Si (59.3 MHz), NMR spectra were recorded on a Varian Unity INOVA 300. Samples for ^29^Si spectra were either dissolved in deuterated solvents or in cases of reaction samples measured with a D_2_O capillary in order to provide an external lock frequency signal. To compensate for the low isotopic abundance of ^29^Si the INEPT pulse sequence [60], [61] was used for the amplification of the signal. If not noted otherwise the used solvent was C_6_D_6_ and all samples were measured at rt. Mass spectra were run on an HP 5971/A/5890-II GC/MS instrument (HP 1 capillary column, length 25 m, diameter 0.2 mm, 0.33 μm poly(dimethylsiloxane)). Elementary analysis was carried using a Heraeus VARIO ELEMENTAR EL apparatus. UV spectra were measured on a Perkin Elmer Lambda 35 spectrometer using spectroscopy grade pentane as solvent.

### 4.2 X-ray structure determination

For X-ray structure analyses the crystals were mounted onto the tip of glass fibers, and data collection was performed with a BRUKER-AXS SMART APEX CCD diffractometer using graphite-monochromated Mo Kα radiation (0.71073 Å). The data were reduced to *F*^2^_o_ and corrected for absorption effects with saint [62] and sadabs,[63], [64] respectively. Structures were solved by direct methods and refined by full-matrix least-squares method (shelxl97 and shelx2013) [65]. All non-hydrogen atoms were refined with anisotropic displacement parameters. Hydrogen atoms were placed in calculated positions to correspond to standard bond lengths and angles. Crystallographic data (excluding structure factors) for the structures of compounds **9**, **11**, **13**, **14**, **15**, **20**, **22**, **23**, **25**, **26**, **27**, and **29** reported in this paper have been deposited with the Cambridge Crystallographic Data Center as supplementary publication no. CCDC-994018 (**9**), 974730 (**11**), 974734 (**13**), 974727 (**14**), 974728 (**15**), 974723 (**20**), 974722 (**22**)**,** 974724 (**23**), 974726 (**25**), 974725 (**26**), 974729 (**27**), and 974733 (**29**). Copies of data can be obtained free of charge at: http://www.ccdc.cam.ac.uk/products/csd/request/.

2,2,5,5-Tetrakis(trimethylsilyl)decamethylhexasilane (**1**) [34], [43], 2,5-bis(trimethylgermyl)-2,5-bis(trimethylsilyl)decamethylhexasilane (**2**) [33], 2,2,5,5-tetrakis(trimethylsilyl)-2,5-digermadecamethylhexasilane (**3**) [33], 2,5-bis(trimethylgermyl)-2,5-bis(trimethylsilyl)-2,5-digermadecamethylhexasilane (**4**) [33], 1,1,1,4,4,4-hexakis(trimethylsilyl)tetramethyltetragermane (**5**) [33], 2,2,5,5-tetrakis(trimethylsilyl)decamethylhexagermane (**6**) [33], dichlorodimethylgermane [66], tris(trimethylsilyl)germyl potassium (**7**) [36], tris(trimethylsilyl)silyl potassium (**28**) [38], [67], 1,3-dichlorohexamethyltrisilane [39], 1,4-dichlorooctamethyltetrasilane [39], 1,5-diphenyldecamethylpentasilane [68], 1,6-dichlorododecamethylhexasilane [39], tris (tri methyl germyl) germyl potassium·18-crown-6 (**18**),[40] 2,2,5,5-tetrakis(trimethylgermyl)octamethylpentagermane (**19**) [40], 1,2-bis[potassiobis(trimethylsilyl)germyl]tetramethyldisilane (**24**) [36], 1,2-dichlorotetramethyldisilane [69], chlorotriisopropylgermane [70], and bromotriisopropylgermane [71] have been prepared following published procedures. All other chemicals were obtained from different suppliers and used without further purification.

#### 4.2.1 Dimethylphenylsilyltris(trimethylsilyl)germane (**8**)

To a solution of dimethylphenylchlorosilane (2.14 g, 12.5 mmol) in THF (20 mL) a solution of tris(trimethylsilyl)germyl potassium (11.7 mmol) in THF (5 mL) was added dropwise. After stirring for 12 h the suspension was added to a mixture of H_2_SO_4_ (0.5 M)/Et_2_O/ice. The layers were separated, the aqueous phase was extracted three times with Et_2_O and from the combined organic layers the solvent was removed in vacuum. The residue was dissolved in pentane, filtered through a pad of silica and then recrystallized from EtOH. Colorless solid **8** was obtained (3.32 g, 66%). Mp.: 169–172 °C. ^1^H NMR (*δ* in ppm): 7.51 (m, 1H), 7.14 (m, 4H), 0.52 (s, 6H), 0.22 (s, 27H). ^13^C NMR (*δ* in ppm): 141.2, 133.9, 128.5, 127.6, 3.2 (Si(*C*H_3_)_3_), 1.5 (Si(*C*H_3_)_2_). ^29^Si NMR (δ in ppm): -5.2 (Me_3_*Si*), −9.4 (Me_2_*Si*). MS: *m*/*z* (%): 428 (19) [M], 340 (12) [M^+^−SiMe_4_], 278 (85) [(SiMe_3_)_2_GeSiMe_2_], 135 (100) [Me_2_PhSi], 73 (98) [SiMe_3_].

#### 4.2.2 Triphenylsilyltris(trimethylsilyl)germane (**9**)

Utilizing a procedure related to the one for the preparation of **8**, tris(trimethylsilyl)germyl potassium [prepared from tetrakis(trimethylsilyl)germane (0.50 g, 1.37 mmol), KO^t^Bu (161 mg, 1.44 mmol), and 18-crown-6 (380 mg, 1.44 mmol)] was reacted with chlorotriphenylsilane (0.42 g, 1.434 mmol). Colorless crystalline **9** was obtained (0.47 g, 80%). Mp.: 295–297 °C. ^1^H NMR (*δ* in ppm, CDCl_3_): 7.50 (m, 6H), 7.364 (m, 9H), 0.19 (s, 27H). ^13^C NMR (*δ* in ppm, CDCl_3_): 136.9, 136.3, 128.9, 127.7, 3.2 (Si(*C*H_3_)_3_). ^29^Si NMR (*δ* in ppm, CDCl_3_): −4.9 (Me_3_Si), −9.4 (Ph_3_Si). *Anal*. Calc. for C_27_H_42_GeSi_4_ 551.60: C, 58.79; H, 7.68. Found: C, 61.64; H, 6.68%. UV absorption: *λ*_1_ = 240 nm (*ε*_1_ = 4.4 × 10^4^ M^−1^ cm^−1^).

#### 4.2.3 Bis[(trimethylsilyl)germyl]dimethylsilane (**11**)

To a solution of **8** (0.75 g, 1.67 mmol) in toluene (10 mL) trifluoromethanesulfonic acid (0.26 g, 1.76 mmol) was added. After 1 h complete formation of triflate **10** was detected by NMR spectroscopy of an aliquot sample (^29^Si NMR of **10** (*δ* in ppm, D_2_O-capillary): 47.1 [Me_2_*Si*OTf], −5.0 [Me_3_*Si*]). A solution of **7** (1.67 mmol) in toluene (10 mL) was added dropwise and after 2 h the solution was poured onto a mixture of H_2_SO_4_ (0.5 M)/Et_2_O/ice. The phases were separated; the aqueous layer extracted three times with Et_2_O, the combined organic phases were dried over Na_2_SO_4_ and the solvent removed in vacuum. Recrystallization from pentane/acetone provided colorless crystals of **11** (0.90 g, 88%). ^1^H NMR (*δ* in ppm): 0.67 (s, 6H), 0.35 (s, 54H). ^13^C NMR (*δ* in ppm): 7.33 ((*C*H_3_)_2_Si), 4.31 ((*C*H_3_)_3_Si). ^29^Si NMR (*δ* in ppm): −4.8 (Me_3_*Si*Ge), −16.3 (Me_2_*Si*). MS: *m*/*z* (%): 351 (100) [M^+^−Ge(SiMe_3_)_3_], 278 (41) [(SiMe_3_)_2_GeSiMe_2_], 203 (19) [Me_3_SiGeSiMe_2_], 131 (22) [Me_2_SiGe], 73 (88) [SiMe_3_]. UV: *λ*_1_ = 245 nm, *ε*_1_ = 4.1 × 10^4^ [M^−1^ cm^−1^].

#### 4.2.4 1,1,1-Tris(trimethylsilyl)-2-tris(trimethylsilyl)germyl-2,2-dimethyldisilane (**12**)

Reaction was done analogously to the preparation of **11** employing: **8** (0.75 g, 1.67 mmol), trifluoromethanesulfonic acid (0.26 g, 1.76 mmol), and tris(trimethylsilyl)silyl potassium (1.67 mmol). Colorless crystals of **12** (0.85 g, 85%) were obtained. ^1^H NMR (*δ* in ppm): 0.63 (s, 6H), 0.35 (s, 27H), 0.32 (s, 27H). ^13^C NMR (*δ* in ppm): 6.5 [Si(*C*H_3_)_2_], 4.4 [SiSi(*C*H_3_)_3_], 3.7 [GeSi(*C*H_3_)_3_]. ^29^Si NMR (*δ* in ppm): −4.6 [Me_3_*Si*Ge], −9.7 [Me_3_*Si*Si], −21.2 [Me_2_*Si*], −119.6 [*Si_q_*]. MS: *m*/*z* (%): 451 (0.5) [M^+^−(SiMe_3_)_2_], 351 (13) [M^+^−Si(SiMe_3_)_3_], 305 (100) [M^+^−Ge(SiMe_3_)_3_], 278 (39) [(SiMe_3_)_2_GeSiMe_2_], 231 (35) [Si(SiMe_3_)_3_−Me], 173 (14) [Si(SiMe_3_)_2_], 73 (62) [SiMe_3_]. UV Absorption: *λ*_1_ = 246 nm, *ε*_1_ = 3.4 × 10^4^ [M^−1^ cm^−1^].

#### 4.2.5 1,1,1,3,3,3-Hexakis(trimethylsilyl)dimethyltrigermane (**13**)

To a solution of Me_2_GeCl_2_ (1.92 g, 11.1 mmol) in THF (20 mL) a solution of **7** (21.1 mmol) in THF (15 mL) was added dropwise at −70 °C. After stirring for 16 h at rt diluted H_2_SO_4_ (0.5 M. 30 mL) was added. The layers were separated; the aqueous layer extracted three times with Et_2_O, the combined organic phases were dried over Na_2_SO_4_ and the solvent removed in vacuum. Pure crystalline **13** (4.16 g, 58%) was obtained after sublimation. Mp.: 219–221 °C. ^1^H NMR (*δ* in ppm): 0.82 (s, 6H), 0.38 (s, 54H). ^13^C NMR (*δ* in ppm): 7.7 (Me_2_Ge), 4.5 (SiMe_3_). ^29^Si NMR (*δ* in ppm): −4.1. UV Absorption: λ _1_ = 253 nm, *ε*_1_ = 4.6 × 10^4^ [M^−1^ cm^−1^]. *Anal*. Calc. for C_20_H_60_Ge_3_Si_6_ (690.09): C, 34.96; H, 8.80. Found: C, 35.23; H, 8.47%.

#### 4.2.6 2,6-Digerma-2,2,6,6-tetrakis(trimethylsilyl)dodecamethylheptasilane (**14**)

Reaction procedure as described for **13**, but at rt using **7** (0.55 mmol) and 1,3-dichlorohexamethyltrisilane (71 mg, 0.29 mmol). After removal of the solvent **14** (150 mg, 77%) was obtained as a colorless solid. Mp.: 175–177 °C. ^1^H NMR (*δ* in ppm): 0.55 (s, 12H, Me_2_Si), 0.50 (s, 6H, Me_2_Si), 0.36 (s, 54H, SiMe_3_). ^13^C NMR (*δ* in ppm): 4.2 (SiMe_3_), 1.7 (SiMe_2_), −2.0 (SiMe_2_). ^29^Si NMR (*δ* in ppm): −5.0 (SiMe_3_), −24.6 (2 × Me_2_Si), −38.1 (Me_2_Si). UV Absorption: *λ*_1_ = 269 nm (*ε*_1_ = 1.0 × 10^5^ [M^−1^ cm^−1^]). *Anal*. Calc. for C_24_H_72_Ge_2_Si_9_ (760.20): C, 37.98; H, 9.56. Found: C, 37.15; H, 9.40%.

#### 4.2.7 2,7-Digerma-2,2,7,7-tetrakis(trimethylsilyl)tetradecamethyloctasilane (**15**)

Reaction procedure as described for **14** using **7** (0.55 mmol) and 1,4-dichlorooctamethyltetrasilane (87 mg, 0.29 mmol). After recrystallization with pentane/acetone colorless crystalline **15** (192 mg, 82%) was obtained. Mp.: 192–195 °C. ^1^H NMR (*δ* in ppm): 0.55 (s, 12H, Me_2_Si), 0.53 (s, 12H, Me_2_Si), 0.36 (s, 54H, SiMe_3_). ^13^C NMR (*δ* in ppm): 4.2 (SiMe_3_), 1.8 (Me_2_Si), −2.7 (Me_2_Si). ^29^Si NMR (*δ* in ppm): −5.1 (SiMe_3_), −25.5 (Me_2_Si), −36.9 (Me_2_Si). UV absorption: *λ*_1_ = 279 nm (*ε*_1_ = 1.0 × 10^5^ [M^−1^ cm^−1^]). *Anal*. Calc. for C_26_H_78_Ge_2_Si_10_ (818.22): C, 38.22; H, 9.62. Found: C, 37.56; H, 9.42%.

#### 4.2.8 2,8-Digerma-2,2,8,8-tetrakis(trimethylsilyl)hexadecamethylnonasilane (**16**)

To a solution of 1,5-bis(trifluoromethanesulfoxyl)decamethylpentasilane (651 mg, 1.05 mmol) [freshly prepared from 1,5-diphenyldecamethylpentasilane (1.05 mmol) and trifluoromethanesulfonic acid (2.20 mmol)] [72] in toluene (5 mL) was slowly added to a solution of **7** in THF (4 mL) at 0 °C. After 14 h the reaction mixture was worked up as described before for **13**. Tetrakis(trimethylsilyl)germane was formed as a side product and could be removed by sublimation yielding pure colorless crystalline **16** (210 mg, 23%). Mp.: 129–131 °C. ^1^H NMR (*δ* in ppm): 0.53 (s, 12H, Me_2_Si), 0.42 (s, 12H, Me_2_Si), 0.39 (s, 6H, Me_2_Si), 0.36 (s, 54H, SiMe_3_). ^13^C NMR (*δ* in ppm): 4.2 (SiMe_3_), 1.7 (2 × Me_2_Si), −2.7 (2 × Me_2_Si), −3.3 (Me_2_Si). ^29^Si NMR (*δ* in ppm): −5.1 (SiMe_3_), -25.7 (2 × Me_2_Si), −36.1 (Me_2_Si), −37.2 (2 × Me_2_Si). UV absorption: *λ*_1_ = 273 nm (*ε*_1_ = 3.0 × 10^4^ [M^−1^ cm^−1^]), λ_2_ = 286 nm (*ε*_2_ = 3.0 × 10^4^ [M^−1^ cm^−1^]). *Anal*. Calc. for C_28_H_84_Ge_2_Si_11_ (876.25): C, 38.43; H, 9.67. Found: C, 38.17; H, 9.47%.

#### 4.2.9 2,9-Digerma-2,2,9,9-tetrakis(trimethylsilyl)octadecamethyldecasilane (**17**)

The reaction was carried out as described for **14** using **7** (1.07 mmol) and 1,6-dichlorododecamethylhexasilane (236 mg, 0.56 mmol). After recrystallization from Et_2_O/acetone colorless crystalline **17** (326 mg, 65%) was obtained. Mp.: 193–195 °C. ^1^H NMR (*δ* in ppm): 0.54 (s, 12H, Me_2_Si), 0.43 (s, 12H, Me_2_Si), 0.39 (s, 12H, Me_2_Si), 0.37 (s, 54H, SiMe_3_). ^13^C NMR (δ in ppm): 4.1 (SiMe_3_), 1.7 (2 × Me_2_Si), −2.8 (2 × Me_2_Si), −3.3 (2 × Me_2_Si). ^29^Si NMR (*δ* in ppm): −5.1 (SiMe_3_), −26.0 (2 × Me_2_Si), −36.6 (2 × Me_2_Si), −37.3 (2 × Me_2_Si). UV absorption: *λ*_1_ = 279 nm (*ε*_1_ = 4.4 × 10^4^ [M^−1^ cm^−1^]), *λ*_2_ = 294 nm (*ε*_2_ = 5.4 × 10^4^ [M^−1^ cm^−1^]). *Anal*. Calc. for C_30_H_90_Ge_2_Si_12_ (934.27): C, 37.58; H, 9.46. Found: C, 37.58; H, 9.13%.

#### 4.2.10 1,2-Bis[tris(trimethylgermy)germyl]tetramethyldisilane (**20**)

The reaction was carried out as described for **14** using **18** (0.28 mmol) and 1,2-dichlorotetramethyldisilane (27 mg, 0.14 mmol). After recrystallization from pentane/acetone colorless crystalline **20** (103 mg, 77%) was obtained. Mp.: 251–253 °C. ^1^H NMR (*δ* in ppm): 0.53 (s, 12H, Me_2_Si), 0.48 (s, 54H, Me_3_Ge). ^13^C NMR (*δ* in ppm): 3.8 (Me_3_Ge), 1.5 (Me_2_Si). ^29^Si NMR (*δ* in ppm): -23.0 (Me_2_Si). UV absorption: *λ*_1_ = 259 nm (*ε*_1_ = 4.7 × 10^4^ [M^−1^ cm^−1^]). *Anal*. Calc. for C_22_H_66_Ge_8_Si_2_ (977.84): C, 27.30; H, 6.87. Found: C, 27.48; H, 6.77%.

#### 4.2.11 1,3-Bis[tris(trimethylgermy)germyl]hexamethyltrisilane (**21**)

The reaction was carried out as described for **14** using **18** (0.28 mmol) and 1,3-dichlorohexamethyltrisilane (36 mg, 0.14 mmol). After recrystallization with cyclohexane colorless crystalline **21** (134 mg, 93%) was obtained. Mp.: 184–186 °C. ^1^H NMR (*δ* in ppm): 0.50 (s, 12H, Me_2_SiGe), 0.48 (s, 54H, Me_3_Ge), 0.46 (s, 6H, Me_2_Si). ^13^C NMR (*δ* in ppm): 3.7 (Me_3_Ge), 1.4 (Me_2_SiGe), −2.7 (Me_2_Si). ^29^Si NMR (*δ* in ppm): −39,3 (Me_2_Si), −22.2 (Me_2_SiGe). UV absorption: λ_1_ = 272 nm (*ε*_1_ = 7.1 × 10^4^ [M^−1^ cm^−1^]). *Anal*. Calc. for C_24_H_72_Ge_8_Si_3_ (1035.86): C, 28.09; H, 7.07. Found: C, 28.05; H, 6.64%.

#### 4.2.12 1,4-Bis[tris(trimethylgermy)germyl]octamethyltetrasilane (**22**)

The reaction was carried out as described for **14** using **18** (0.37 mmol) and 1,4-dichlorooctamethyltetrasilane (59 mg, 0.19 mmol). After recrystallization from pentane/acetone **22** (150 mg, 75%) was obtained as colorless crystals. Mp.: 179–181 °C. ^1^H NMR (*δ* in ppm): 0.50 (s, 12H, Me_2_SiGe), 0.50 (s, 54H, Me_3_Ge), 0.38 (s, 12H, Me_2_Si). ^13^C NMR (*δ* in ppm): 3.7 (Me_3_Ge), 1.4 (Me_2_SiGe), −3.2 (Me_2_Si). ^29^Si NMR (*δ* in ppm): −22.5 (Me_2_SiGe), −37.8 (Me_2_Si). UV absorption: *λ*_1_ = 282 nm (*ε*_1_ = 7.4 × 10^4^ [M^−1^ cm^−1^]). *Anal*. Calc. for C_26_H_78_Ge_8_Si_4_ (1093.89): C, 28.80; H, 7.25. Found: C, 28.91; H, 7.18%.

#### 4.2.13 1,6-Bis[tris(trimethylgermy)germyl]dodecamethylhexasilane (**23**)

The reaction was carried out as described for **14** using **18** (0.28 mmol) and 1,6-dichlorododecamethylhexasilane (61 mg, 0.14 mmol). After recrystallization with cyclohexane colorless crystalline **23** (167 mg, 99%) was obtained. ^1^H NMR (*δ* in ppm): 0.52 (s, 12H, Me_2_Si), 0.50 (s, 54H, Me_3_Ge), 0.40 (s, 12H, Me_2_Si), 0.36 (s, 12H, Me_2_Si). ^13^C NMR (*δ* in ppm): 3.7 (Me_3_Ge), 1.4 (Me_2_Si), −3.2 (Me_2_Si), -3,5 (Me_2_Si). ^29^Si NMR (*δ* in ppm): −22,8 (Me_2_Si), −36.9 (Me_2_Si), −38.1 (Me_2_Si). UV absorption: *λ*_1_ = 251 nm (*ε*_1_ = 2.5 × 10^4^ [M^−1^ cm^−1^]), *λ*_2_ = 282 nm (*ε*_2_ = 7.4 × 10^4^ [M^−1^ cm^−1^], shoulder), *λ*_3_ = 296 nm (*ε*_3_ = 5.8 × 10^4^ [M^−1^ cm^−1^]).

#### 4.2.14 1,4-Digerma-1,1,4,4-tetrakis(trimethylsilyl)octamethylcyclohexasilane (**25**)

The reaction was carried out as described for **14** using DME as a solvent, **24** (0.28 mmol) and 1,2-dichlorotetramethyldisilane (39 mg, 0.30 mmol). After recrystallization from pentane/acetone colorless crystalline **25** (140 mg, 80%) was obtained. Mp.: 152–154 °C. ^1^H NMR (*δ* in ppm): 0.44 (s, 24H, Me_2_Si), 0.36 (s, 36H, SiMe_3_). ^13^C NMR (*δ* in ppm): 4.4 (SiMe_3_), −0.3 (Me_2_Si). ^29^Si NMR (*δ* in ppm): −3.6 (SiMe_3_), −32.8 (Me_2_Si). *Anal*. Calc. for C_20_H_60_Ge_2_Si_8_ (670,65): C, 35.82; H, 9.02. Found: C, 36.07; H, 8.56%.

#### 4.2.15 1,3-Digerma-1,1,3,3-tetrakis(trimethylsilyl)hexamethylcyclopentasilane (**26**)

The reaction was carried out as described for **14** using **24** (0.29 mmol) and dichlorodimethylsilane (39 mg, 0.30 mmol). After recrystallization from pentane/acetone colorless crystalline **26** (140 mg, 80%) was obtained. ^1^H NMR (*δ* in ppm): 0.65 (s, 6H, GeSiMe_2_Ge), 0.43 (s, 12H, GeSiMe_2_SiMe_2_), 0.34 (s, 36H, SiMe_3_). ^13^C NMR (*δ* in ppm): 5.6 (GeSiMe_2_Ge), 4.4 (SiMe_3_), *−*1.2 (GeSiMe_2_SiMe_2_). ^29^Si NMR (*δ* in ppm): −2.9 (SiMe_3_), −10.1 (GeSiMe_2_Ge), −18.7 (GeSiMe_2_SiMe_2_). *Anal*. Calc. for C_18_H_54_Ge_2_Si_7_ (612.50): C, 35.30; H, 8.89. Found: C, 35.67; H, 8.21%.

#### 4.2.16 Triisopropylgermyltris(trimethylgermyl)germane (**27**)

To a solution of bromotriisopropylgermane (296 mg, 1.05 mmol) in toluene (5 mL) a solution of **18** (1.00 mmol) in toluene (10 mL) was slowly added dropwise and stirred for 2 h. Workup as described for **13** and recrystallization with Et_2_O/acetone yielded colorless crystals of **27** (570 mg, 91%). ^1^H NMR (*δ* in ppm): 1.51 (m, 3H, *CH*CH_3_), 1.22 (d, 18H, *J *= 7 Hz, CH*CH_3_*), 0.50 (s, 27H, SiMe_3_). ^13^C NMR (*δ* in ppm): 21.6 (CH*CH_3_*), 18.7 (*CH*CH_3_), 4.3 (SiMe_3_).

#### 4.2.17 Tris(trimethylsilyl)triisopropylgermylsilane (**29**)

A solution of **28** [prepared from tetrakis(trimethylsilyl)silane (938 mg, 2.92 mmol) and potassium *tert*-butanolate (3.07 mmol)] in THF (20 mL) was added dropwise over a period of 6 h to a solution of chlorotriisopropylgermane (839 mg, 2.98 mmol) in THF (10 mL). After 12 h H_2_SO_4_ (2 M, 10 mL) was added, the layers were separated and the organic layer dried with Na_2_SO_4_. After removal of the solvent **29** (1.20 g, 92%) was obtained as a colorless solid. Mp.: 266–268 °C. ^1^H NMR (*δ* in ppm): 1.53 (m, 3H), 1.23 (d, *J *= 7 Hz, 18H), 0.30 (s, 27H). ^13^C NMR (*δ* in ppm): 21.4 (SiCH(*C*H_3_)_2_); 17.8 (Si*C*H_2_Me_2_); 3.8 (Si(*C*H_3_)_3_). ^29^Si NMR (*δ* in ppm): −9.21 (Me_3_*Si*Si), −115.75 (Ge*Si*Si_3_). *Anal*. Calc. for C_18_H_48_GeSi_4_ (449.55): C, 48.09; H, 10.76. Found: C, 47.51; H, 10.32%.

## Author contributions

The manuscript was written through contributions of all authors. All authors have given approval to the final version of the manuscript. The authors declare no competing financial interest.

## References

[1] Brook M.A. (2000).

[2] C. Marschner, J. Baumgartner, in: M. Oestreich (Ed.), Science of Synthesis: Houben-Weyl Methods of Molecular Transformations. Thieme, Stuttgart, 2013.

[3] Michl J., West R. (2000). Acc. Chem. Res..

[4] Bande A., Michl J. (2009). Chem. Eur. J..

[5] Tsuji H., Michl J., Tamao K. (2003). J. Organomet. Chem..

[6] Wallner A., Emanuelsson R., Baumgartner J., Marschner C., Ottosson H. (2013). Organometallics.

[7] Fukazawa A., Tsuji H., Tamao K. (2006). J. Am. Chem. Soc..

[8] Tsuji H., Fukazawa A., Yamaguchi S., Toshimitsu A., Tamao K. (2004). Organometallics.

[9] Tamao K., Tsuji H., Terada M., Asahara M., Yamaguchi S., Toshimitsu A. (2000). Angew. Chem., Int. Ed..

[10] Mallesha H., Tsuji H., Tamao K. (2004). Organometallics.

[11] Tsuji H., Terada M., Toshimitsu A., Tamao K. (2003). J. Am. Chem. Soc..

[12] Marschner C., Baumgartner J., Wallner A. (2006). Dalton Trans..

[13] Wallner A., Wagner H., Baumgartner J., Marschner C., Rohm H.W., Kockerling M., Krempner C. (2008). Organometallics.

[14] Wallner A., Hlina J., Wagner H., Baumgartner J., Marschner C. (2011). Organometallics.

[15] Tachibana H., Tokura Y. (2005). Synth. Met..

[16] Mochida K., Nagano S. (1998). Inorg. Chem. Commun..

[17] Mustafa A., Achilleos M., Ruiz-Iban J., Davies J., Benfield R.E., Jones R.G., Grandjean D., Holder S.J. (2006). React. Funct. Polym..

[18] Subashi E., Rheingold A.L., Weinert C.S. (2006). Organometallics.

[19] Amadoruge M.L., DiPasquale A.G., Rheingold A.L., Weinert C.S. (2008). J. Organomet. Chem..

[20] Amadoruge M.L., Golen J.A., Rheingold A.L., Weinert C.S. (1979). Organometallics.

[21] Weinert C.S. (2009). Dalton Trans..

[22] Weinert C.S. (2011). Comment. Inorg. Chem..

[23] Samanamu C.R., Amadoruge M.L., Yoder C.H., Golen J.A., Moore C.E., Rheingold A.L., Materer N.F., Weinert C.S. (2011). Organometallics.

[24] Samanamu C.R., Amadoruge M.L., Schrick A.C., Chen C., Golen J.A., Rheingold A.L., Materer N.F., Weinert C.S. (2012). Organometallics.

[25] Amadoruge M.L., Short E.K., Moore C., Rheingold A.L., Weinert C.S. (1813). J. Organomet. Chem..

[26] Amadoruge M.L., Gardinier J.R., Weinert C.S. (2008). Organometallics.

[27] Roewe K.D., Rheingold A.L., Weinert C.S. (2013). Chem. Commun..

[28] Marschner C. (2006). Organometallics.

[29] C. Marschner, in: D. Scheschkewitz (Ed.), Functional molecular silicon compounds I, Springer, New York, 2014, p. 163.

[30] Fischer J., Baumgartner J., Marschner C. (2005). Science.

[31] Wagner H., Baumgartner J., Marschner C., Poelt P. (2011). Organometallics.

[32] Wagner H., Wallner A., Fischer J., Flock M., Baumgartner J., Marschner C. (2007). Organometallics.

[33] Wagner H., Baumgartner J., Müller T., Marschner C. (2009). J. Am. Chem. Soc..

[34] Whittaker S.M., Brun M.-C., Cervantes-Lee F., Pannell K.H. (1995). J. Organomet. Chem..

[35] Lambert J.B., Pflug J.L., Allgeier A.M., Campbell D.J., Higgins T.B., Singewald E.T., Stern C.L. (1995). Acta Crystallogr., Sect. C.

[36] Fischer J., Baumgartner J., Marschner C. (2005). Organometallics.

[37] Uhlig W. (1996). Chem. Ber..

[38] Marschner C. (1998). Eur. J. Inorg. Chem..

[39] Gilman H., Inoue S. (1964). J. Org. Chem..

[40] Hlina J., Baumgartner J., Marschner C. (2010). Organometallics.

[41] Fischer R., Konopa T., Ully S., Baumgartner J., Marschner C. (2003). J. Organomet. Chem..

[42] Kayser C., Kickelbick G., Marschner C. (2002). Angew. Chem., Int. Ed..

[43] Fischer R., Frank D., Gaderbauer W., Kayser C., Mechtler C., Baumgartner J., Marschner C. (2003). Organometallics.

[44] Zaitsev K.V., Kapranov A.A., Churakov A.V., Poleshchuk O.K., Oprunenko Y.F., Tarasevich B.N., Zaitseva G.S., Karlov S.S. (2013). Organometallics.

[45] Samanamu C.R., Materer N.F., Weinert C.S. (2012). J. Organomet. Chem..

[46] Mochida K., Kanno N., Kato R., Kotani M., Yamauchi S., Wakasa M., Hayashi H. (1991). J. Organomet. Chem..

[47] Mochida K., Yoneda I., Wakasa M. (1990). J. Organomet. Chem..

[48] Castel A., Riviere P., Saint-Roch B., Satge J., Malrieu J.P. (1983). J. Organomet. Chem..

[49] Mochida K., Hata R., Shimoda M., Matsumoto F., Kurosu H., Kojima A., Yoshikawa M., Masuda S., Harada Y. (1996). Polyhedron.

[50] Mochida K., Chiba H., Okano M. (1991). Chem. Lett..

[51] Okano M., Mochida K. (1990). Chem. Lett..

[52] Ohshita J., Nekoda E., Masaoka S., Ishikawa M. (1997). J. Organomet. Chem..

[53] Mallela S.P., Ghuman M.A., Geanangel R.A. (1992). Inorg. Chim. Acta.

[54] Baumgartner J., Frank D., Kayser C., Marschner C. (2005). Organometallics.

[55] West R. (2003). J. Organomet. Chem..

[56] Roller S., Simon D., Dräger M. (1986). J. Organomet. Chem..

[57] Fischer J., Gaderbauer W., Baumgartner J., Marschner C. (2006). Heterocycles.

[58] R. Fischer, T. Konopa, S. Ully, A. Wallner, J. Baumgartner, C. Marschner, in: N. Auner, J. Weis (Eds.), Organosilicon Chemistry VI, Wiley-VCH, Weinheim, 2005, p. 355.

[59] Pangborn A.B., Giardello M.A., Grubbs R.H., Rosen R.K., Timmers F.J. (1996). Organometallics.

[60] Morris G.A., Freeman R. (1979). J. Am. Chem. Soc..

[61] Helmer B.J., West R. (1982). Organometallics.

[62] saintplus: Software Reference Manual, Version 6.45, Bruker-AXS, Madison, WI, 1997–2003.

[63] G.M. Sheldrick, sadabs, Version 2.10. Bruker AXS Inc., Madison, USA, 2003.

[64] Blessing R.H. (1995). Acta Crystallogr., Sect. A.

[65] Sheldrick G.M. (2007). Acta Crystallogr., Sect. A.

[66] Bordeau M., Djamei S.M., Dunogues J. (1985). Organometallics.

[67] Kayser C., Fischer R., Baumgartner J., Marschner C. (2002). Organometallics.

[68] Ito Y., Suginome M., Matsuura T., Murakami M. (1991). J. Am. Chem. Soc..

[69] Ishikawa M., Kumada M., Sakurai H. (1970). J. Organomet. Chem..

[70] Mendelsohn J.-C., Metras F., Lahournère J.-C., Valade J. (1968). J. Organomet. Chem..

[71] Anderson H.H. (1953). J. Am. Chem. Soc..

[72] Ruehl K.E., Matyjaszewski K. (1991). J. Organomet. Chem..

