# Supplementary material for: σ-Bond electron delocalization of branched oligogermanes and germanium containing oligosilanes
Source: Inorganica Chim Acta. 2014 Oct 1;422:120–33. doi: 10.1016/j.ica.2014.07.005 (PMC4236089; doi:10.1016/j.ica.2014.07.005)

checkCIF/PLATON report

You have not supplied any structure factors. As a result the full set of tests cannot be run.

THIS REPORT IS FOR GUIDANCE ONLY. IF USED AS PART OF A REVIEW PROCEDURE FOR PUBLICATION, IT SHOULD NOT REPLACE THE EXPERTISE OF AN EXPERIENCED CRYSTALLOGRAPHIC REFEREE.

No syntax errors found. CIF dictionary Interpreting this report

Datablock: 9_1056j

```
Bond precision:  C-C = 0.0040 A          Wavelength=0.71073

Cell:             a=16.881(3)      b=9.822(2)         c=36.650(7)
                  alpha=90         beta=91.48(3)      gamma=90
Temperature:      100 K
```

	Calculated	Reported
Volume	6075(2)	6075(2)
Space group	C 2/c	C2/c
Hall group	-C 2yc	?
Moiety formula	C27 H42 Ge Si4	?
Sum formula	C27 H42 Ge Si4	C27 H42 Ge Si4
Mr	551.58	551.56
Dx,g cm-3	1.206	1.206
Z	8	8
Mu (mm-1)	1.179	1.180
F000	2336.0	2336.0
F000’	2340.18	
h,k,lmax	21,12,45	21,12,45
Nref	6212	6196
Tmin,Tmax	0.679,0.771	0.676,0.781
Tmin’	0.647	

```
Correction method= MULTI-SCAN

Data completeness= 0.997          Theta(max)= 26.370

R(reflections)= 0.0445( 5747)     wR2(reflections)= 0.0995( 6196)

S = 1.145                   Npar= Npar = 298
```

The following ALERTS were generated. Each ALERT has the format
test-name_ALERT_alert-type_alert-level.
Click on the hyperlinks for more details of the test.

Alert level C

```
PLAT094_ALERT_2_C Ratio of Maximum / Minimum Residual Density ....       2.40 Why ?
```

Alert level G

```
PLAT005_ALERT_5_G No _iucr_refine_instructions_details  in the CIF     Please Do !
PLAT083_ALERT_2_G SHELXL Second Parameter in WGHT Unusually Large.      10.56 Why ?
```

---

0 **ALERT level A** = Most likely a serious problem - resolve or explain
0 **ALERT level B** = A potentially serious problem, consider carefully
1 **ALERT level C** = Check. Ensure it is not caused by an omission or oversight
2 **ALERT level G** = General information/check it is not something unexpected

0 ALERT type 1 CIF construction/syntax error, inconsistent or missing data
2 ALERT type 2 Indicator that the structure model may be wrong or deficient
0 ALERT type 3 Indicator that the structure quality may be low
0 ALERT type 4 Improvement, methodology, query or suggestion
1 ALERT type 5 Informative message, check

Datablock: 10_845j

Bond precision: Si- C = 0.0040 A   Wavelength=0.71073

Cell:	a=17.055(3)	b=9.2847(19)	c=24.665(5)
	alpha=90	beta=107.01(3)	gamma=90
Temperature:	240 K		

	Calculated	Reported
Volume	3734.9(14)	3734.7(13)
Space group	C 2/c	C2/c
Hall group	-C 2yc	?
Moiety formula	C20 H60 Ge2 Si7	?
Sum formula	C20 H60 Ge2 Si7	C20 H60 Ge2 Si7
Mr	642.53	642.49
Dx,g cm-3	1.143	1.143
Z	4	4
Mu (mm-1)	1.842	1.842
F000	1368.0	1368.0
F000’	1371.56	
h,k,lmax	21,11,30	21,11,30
Nref	3813	3811
Tmin,Tmax	0.487,0.759	0.526,0.770
Tmin’	0.474	

Correction method= MULTI-SCAN

Data completeness= 0.999   Theta(max)= 26.360

```
R(reflections)= 0.0326( 3253)     wR2(reflections)= 0.0872( 3811)

S = 1.052                   Npar= Npar = 173
```

The following ALERTS were generated. Each ALERT has the format
test-name_ALERT_alert-type_alert-level.
Click on the hyperlinks for more details of the test.

Alert level B

```
PLAT213_ALERT_2_B Atom C66             has ADP max/min Ratio .....        4.2 prolat
PLAT242_ALERT_2_B Low       Ueq as Compared to Neighbors for .....        Si2 Check
PLAT412_ALERT_2_B Short Intra XH3 .. XHn     H8B    ..  H55B    ..       1.72 Ang.
```

Alert level C

```
PLAT094_ALERT_2_C Ratio of Maximum / Minimum Residual Density ....       2.41 Why ?
PLAT213_ALERT_2_C Atom C6              has ADP max/min Ratio .....        3.1 prolat
PLAT213_ALERT_2_C Atom C44             has ADP max/min Ratio .....        3.7 prolat
PLAT242_ALERT_2_C Low       Ueq as Compared to Neighbors for .....        Si1 Check
PLAT242_ALERT_2_C Low       Ueq as Compared to Neighbors for .....        Si3 Check
PLAT242_ALERT_2_C Low       Ueq as Compared to Neighbors for .....        Si4 Check
PLAT412_ALERT_2_C Short Intra XH3 .. XHn     H5B    ..  H7A     ..       1.88 Ang.
```

Alert level G

```
PLAT005_ALERT_5_G No _iucr_refine_instructions_details  in the CIF     Please Do !
PLAT301_ALERT_3_G Main Residue  Disorder ............ Percentage =         21 Note
PLAT779_ALERT_4_G Suspect or Irrelevant (Bond) Angle in CIF .... #         16 Check
              C44  -SI2  -C5      1.555   1.555   1.555            40.90 Deg.
PLAT779_ALERT_4_G Suspect or Irrelevant (Bond) Angle in CIF .... #         20 Check
              C6   -SI2  -C55     1.555   1.555   1.555            44.00 Deg.
PLAT811_ALERT_5_G No ADDSYM Analysis: Too Many Excluded Atoms ....          ! Info
```

```
   0 ALERT level A = Most likely a serious problem - resolve or explain
   3 ALERT level B = A potentially serious problem, consider carefully
   7 ALERT level C = Check. Ensure it is not caused by an omission or oversight
   5 ALERT level G = General information/check it is not something unexpected

   0 ALERT type 1 CIF construction/syntax error, inconsistent or missing data
  10 ALERT type 2 Indicator that the structure model may be wrong or deficient
   1 ALERT type 3 Indicator that the structure quality may be low
   2 ALERT type 4 Improvement, methodology, query or suggestion
   2 ALERT type 5 Informative message, check
```

Datablock: 12_649j

```
Bond precision:   Si- C = 0.0077 A        Wavelength=0.71073

Cell:             a=16.971(3)     b=9.1675(18)       c=24.282(5)
                  alpha=90        beta=106.44(3)     gamma=90
Temperature:      100 K
```

	Calculated	Reported
Volume	3623.4(13)	3623.6(13)
Space group	C 2/c	C2/c
Hall group	-C 2yc	?
Moiety formula	C20 H60 Ge3 Si6	?
Sum formula	C20 H60 Ge3 Si6	C20 H60 Ge3 Si6
Mr	687.05	686.99
Dx,g cm-3	1.260	1.259
Z	4	4
Mu (mm-1)	2.679	2.679
F000	1440.0	1440.0
F000'	1443.87	
h,k,lmax	21,11,30	20,11,30
Nref	3694	3575
Tmin,Tmax	0.418,0.669	0.463,0.689
Tmin'	0.387	

Correction method= MULTI-SCAN

Data completeness= 0.968 Theta(max)= 26.350

R(reflections)= 0.0566(2438) wR2(reflections)= 0.1271(3575)

S = 0.926 Npar= Npar = 167

The following ALERTS were generated. Each ALERT has the format
test-name_ALERT_alert-type_alert-level.
Click on the hyperlinks for more details of the test.

Alert level B

```
PLAT213_ALERT_2_B Atom C1              has ADP max/min Ratio .....        4.4 prolat
PLAT412_ALERT_2_B Short Intra XH3 .. XHn     H8B    ..  H55B    ..       1.70 Ang.
```

Alert level C

```
PLAT029_ALERT_3_C _diffrn_measured_fraction_theta_full Low .......      0.968 Note
PLAT213_ALERT_2_C Atom C8              has ADP max/min Ratio .....        3.6 prolat
PLAT213_ALERT_2_C Atom C9              has ADP max/min Ratio .....        3.2 prolat
PLAT213_ALERT_2_C Atom C10             has ADP max/min Ratio .....        3.4 prolat
PLAT220_ALERT_2_C Large Non-Solvent  C     Ueq(max)/Ueq(min) Range        3.7 Ratio
PLAT242_ALERT_2_C Low       Ueq as Compared to Neighbors for .....        Si1 Check
PLAT242_ALERT_2_C Low       Ueq as Compared to Neighbors for .....        Si3 Check
```

Alert level G

```
PLAT005_ALERT_5_G No _iucr_refine_instructions_details  in the CIF     Please Do !
PLAT301_ALERT_3_G Main Residue  Disorder ............ Percentage =         21 Note
PLAT779_ALERT_4_G Suspect or Irrelevant (Bond) Angle in CIF .... #         21 Check
              C44  -SI2  -C4      1.555   1.555   1.555              34.30 Deg.
PLAT779_ALERT_4_G Suspect or Irrelevant (Bond) Angle in CIF .... #         29 Check
              C5   -SI2  -C55     1.555   1.555   1.555              40.30 Deg.
PLAT811_ALERT_5_G No ADDSYM Analysis: Too Many Excluded Atoms ....          ! Info
```

```
   0 ALERT level A = Most likely a serious problem - resolve or explain
   2 ALERT level B = A potentially serious problem, consider carefully
   7 ALERT level C = Check. Ensure it is not caused by an omission or oversight
   5 ALERT level G = General information/check it is not something unexpected

   0 ALERT type 1 CIF construction/syntax error, inconsistent or missing data
   8 ALERT type 2 Indicator that the structure model may be wrong or deficient
   2 ALERT type 3 Indicator that the structure quality may be low
   2 ALERT type 4 Improvement, methodology, query or suggestion
   2 ALERT type 5 Informative message, check
```

Datablock: 13_729j

```
Bond precision:  Si- C = 0.0086 A       Wavelength=0.71073

Cell:            a=15.585(3)    b=9.899(2)      c=58.109(12)
                 alpha=90       beta=96.19(3)   gamma=90
Temperature:     100 K
```

	Calculated	Reported
Volume	8913(3)	8913(3)
Space group	C 2/c	C2/c
Hall group	-C 2yc	?
Moiety formula	C24 H72 Ge2 Si9	?
Sum formula	C24 H72 Ge2 Si9	C24 H72 Ge2 Si9
Mr	758.85	758.81
Dx,g cm-3	1.131	1.131
Z	8	8
Mu (mm-1)	1.604	1.604
F000	3248.0	3248.0
F000’	3256.46	
h,k,lmax	18,11,69	18,11,68
Nref	7814	7812
Tmin,Tmax	0.624,0.749	0.581,0.761
Tmin’	0.538	

```
Correction method= MULTI-SCAN

Data completeness= 1.000          Theta(max)= 25.000

R(reflections)= 0.0930( 6981)     wR2(reflections)= 0.1788( 7812)

S = 1.343                  Npar= Npar = 340
```

The following ALERTS were generated. Each ALERT has the format
test-name_ALERT_alert-type_alert-level.
Click on the hyperlinks for more details of the test.

Alert level G

```
PLAT005_ALERT_5_G No _iucr_refine_instructions_details  in the CIF     Please Do !
PLAT083_ALERT_2_G SHELXL Second Parameter in WGHT Unusually Large.     147.72 Why ?
```

```
   0 ALERT level A = Most likely a serious problem - resolve or explain
   0 ALERT level B = A potentially serious problem, consider carefully
   0 ALERT level C = Check. Ensure it is not caused by an omission or oversight
   2 ALERT level G = General information/check it is not something unexpected

   0 ALERT type 1 CIF construction/syntax error, inconsistent or missing data
   1 ALERT type 2 Indicator that the structure model may be wrong or deficient
   0 ALERT type 3 Indicator that the structure quality may be low
   0 ALERT type 4 Improvement, methodology, query or suggestion
   1 ALERT type 5 Informative message, check
```

Datablock: 14_760j

```
Bond precision:  Si- C = 0.0028 A        Wavelength=0.71073

Cell:           a=8.9902(18)      b=9.1616(18)     c=16.368(3)
                alpha=82.10(3)    beta=75.60(3)    gamma=66.72(3)
Temperature:    100 K

                Calculated                 Reported
Volume          1198.3(5)                  1198.4(4)
Space group     P -1                       P-1
Hall group      -P 1                       ?
Moiety formula  C26 H78 Ge2 Si10           ?
Sum formula     C26 H78 Ge2 Si10           C26 H78 Ge2 Si10
Mr              817.00                     816.96
Dx,g cm-3       1.132                      1.132
Z               1                          1
Mu (mm-1)       1.519                      1.519
F000            438.0                      438.0
F000'           439.14
h,k,lmax        11,11,20                   11,11,20
Nref            4896                       4814
Tmin,Tmax       0.606,0.833                0.596,0.839
Tmin'           0.556

Correction method= MULTI-SCAN

Data completeness= 0.983          Theta(max)= 26.360

R(reflections)= 0.0299( 4492)     wR2(reflections)= 0.0720( 4814)

S = 1.036                   Npar= Npar = 185
```

The following ALERTS were generated. Each ALERT has the format
test-name_ALERT_alert-type_alert-level.
Click on the hyperlinks for more details of the test.

Alert level C

PLAT094_ALERT_2_C Ratio of Maximum / Minimum Residual Density .... 2.71 Why ?

Alert level G

PLAT005_ALERT_5_G No _iucr_refine_instructions_details in the CIF Please Do !
PLAT154_ALERT_1_G The su's on the Cell Angles are Equal .... 0.03000 Degree

0 **ALERT level A** = Most likely a serious problem - resolve or explain
0 **ALERT level B** = A potentially serious problem, consider carefully
1 **ALERT level C** = Check. Ensure it is not caused by an omission or oversight
2 **ALERT level G** = General information/check it is not something unexpected

1 ALERT type 1 CIF construction/syntax error, inconsistent or missing data
1 ALERT type 2 Indicator that the structure model may be wrong or deficient
0 ALERT type 3 Indicator that the structure quality may be low
0 ALERT type 4 Improvement, methodology, query or suggestion
1 ALERT type 5 Informative message, check

Datablock: 19_661j

Bond precision: Ge- C = 0.0073 A Wavelength=0.71073

Cell:	a=16.022(3)	b=9.915(2)	c=26.387(5)
	alpha=90	beta=92.34(3)	gamma=90
Temperature:	100 K		

	Calculated	Reported
Volume	4188.3(14)	4188.3(15)
Space group	C 2/c	C2/c
Hall group	-C 2yc	?
Moiety formula	C22 H66 Ge8 Si2	?
Sum formula	C22 H66 Ge8 Si2	C22 H66 Ge8 Si2
Mr	967.81	967.65
Dx,g cm-3	1.535	1.535
Z	4	4
Mu (mm-1)	5.726	5.726
F000	1928.0	1928.0
F000'	1933.95	
h,k,lmax	20,12,32	20,12,32
Nref	4290	4269
Tmin,Tmax	0.196,0.503	0.278,0.547
Tmin'	0.165	

Correction method= MULTI-SCAN

Data completeness= 0.995 Theta(max)= 26.370

R(reflections)= 0.0593(3589) wR2(reflections)= 0.1124(4269)

S = 1.215 Npar= Npar = 156

The following ALERTS were generated. Each ALERT has the format
test-name_ALERT_alert-type_alert-level.
Click on the hyperlinks for more details of the test.

Alert level B

```
PLAT230_ALERT_2_B Hirshfeld Test Diff for    Ge1    --  Ge2     ..        7.8 su
PLAT230_ALERT_2_B Hirshfeld Test Diff for    Ge1    --  Ge3     ..       11.6 su
PLAT230_ALERT_2_B Hirshfeld Test Diff for    Ge1    --  Ge4     ..        8.8 su
```

Alert level C

```
PLAT230_ALERT_2_C Hirshfeld Test Diff for    Ge1    --  Si1     ..        5.4 su
```

Alert level G

```
PLAT005_ALERT_5_G No _iucr_refine_instructions_details  in the CIF     Please Do !
PLAT083_ALERT_2_G SHELXL Second Parameter in WGHT Unusually Large.      26.48 Why ?
```

---

0 **ALERT level A** = Most likely a serious problem - resolve or explain
3 **ALERT level B** = A potentially serious problem, consider carefully
1 **ALERT level C** = Check. Ensure it is not caused by an omission or oversight
2 **ALERT level G** = General information/check it is not something unexpected

0 ALERT type 1 CIF construction/syntax error, inconsistent or missing data
5 ALERT type 2 Indicator that the structure model may be wrong or deficient
0 ALERT type 3 Indicator that the structure quality may be low
0 ALERT type 4 Improvement, methodology, query or suggestion
1 ALERT type 5 Informative message, check

Datablock: 21_660j

Bond precision: Si- C = 0.0055 A Wavelength=0.71073

Cell:	a=9.0562(18)	b=9.2540(19)	c=16.465(3)
	alpha=82.49(3)	beta=76.48(3)	gamma=67.77(3)
Temperature:	150 K		

	Calculated	Reported
Volume	1240.5(5)	1240.5(4)
Space group	P -1	P-1
Hall group	-P 1	?
Moiety formula	C26 H78 Ge8 Si4	?
Sum formula	C26 H78 Ge8 Si4	C26 H78 Ge8 Si4
Mr	1084.12	1083.96
Dx,g cm-3	1.451	1.451
Z	1	1
Mu (mm-1)	4.887	4.887
F000	546.0	546.0
F000’	547.63	
h,k,lmax	11,11,20	11,11,20
Nref	5026	4940
Tmin,Tmax	0.148,0.231	0.233,0.322
Tmin’	0.112	

Correction method= MULTI-SCAN

Data completeness= 0.983 Theta(max)= 26.290

R(reflections)= 0.0421(4125) wR2(reflections)= 0.1085(4940)

S = 1.022 Npar= Npar = 185

The following ALERTS were generated. Each ALERT has the format
test-name_ALERT_alert-type_alert-level.
Click on the hyperlinks for more details of the test.

Alert level B

PLAT230_ALERT_2_B Hirshfeld Test Diff for Ge1 -- Ge2 .. 13.8 su
PLAT230_ALERT_2_B Hirshfeld Test Diff for Ge1 -- Ge3 .. 22.3 su
PLAT230_ALERT_2_B Hirshfeld Test Diff for Ge1 -- Ge4 .. 21.0 su
PLAT230_ALERT_2_B Hirshfeld Test Diff for Ge1 -- Si1 .. 10.0 su

---

Alert level C

PLAT094_ALERT_2_C Ratio of Maximum / Minimum Residual Density .... 2.40 Why ?

Alert level G

PLAT005_ALERT_5_G No _iucr_refine_instructions_details in the CIF Please Do !
PLAT154_ALERT_1_G The su’s on the Cell Angles are Equal .... 0.03000 Degree

---

0 **ALERT level A** = Most likely a serious problem - resolve or explain
4 **ALERT level B** = A potentially serious problem, consider carefully
1 **ALERT level C** = Check. Ensure it is not caused by an omission or oversight
2 **ALERT level G** = General information/check it is not something unexpected

1 ALERT type 1 CIF construction/syntax error, inconsistent or missing data

```
5 ALERT type 2 Indicator that the structure model may be wrong or deficient
0 ALERT type 3 Indicator that the structure quality may be low
0 ALERT type 4 Improvement, methodology, query or suggestion
1 ALERT type 5 Informative message, check
```

Datablock: 22_672j

Bond precision: Si- C = 0.0143 A   Wavelength=0.71073

Cell:	a=14.566(3)	b=9.7242(19)	c=41.632(8)
	alpha=90	beta=94.27(3)	gamma=90
Temperature:	136 K		

	Calculated	Reported
Volume	5881(2)	5881(2)
Space group	P 21/c	P2(1)/c
Hall group	-P 2ybc	?
Moiety formula	C30 H90 Ge8 Si6	?
Sum formula	C30 H90 Ge8 Si6	C30 H90 Ge8 Si6
Mr	1200.45	1200.28
Dx,g cm-3	1.356	1.356
Z	4	4
Mu (mm-1)	4.169	4.170
F000	2440.0	2440.0
F000'	2447.14	
h,k,lmax	17,11,49	17,11,49
Nref	10368	10362
Tmin,Tmax	0.382,0.606	0.331,0.635
Tmin'	0.233	

Correction method= MULTI-SCAN

Data completeness= 0.999   Theta(max)= 25.000

R(reflections)= 0.0962(5691)   wR2(reflections)= 0.1697(10362)

S = 1.087   Npar= Npar = 427

The following ALERTS were generated. Each ALERT has the format
test-name_ALERT_alert-type_alert-level.
Click on the hyperlinks for more details of the test.

Alert level B

PLAT230_ALERT_2_B Hirshfeld Test Diff for   Ge1   --   Ge2   ..   7.5 su

Alert level C

RINTA01_ALERT_3_C The value of Rint is greater than 0.12

```
          Rint given   0.162
PLAT020_ALERT_3_C The value of Rint is greater than 0.12 .........      0.162
PLAT230_ALERT_2_C Hirshfeld Test Diff for    Ge5    --  Ge7     ..        6.5 su
PLAT234_ALERT_4_C Large Hirshfeld Difference Si4    --  C26     ..       0.21 Ang.
PLAT242_ALERT_2_C Low       Ueq as Compared to Neighbors for .....        Ge1 Check
PLAT242_ALERT_2_C Low       Ueq as Compared to Neighbors for .....        Ge2 Check
PLAT242_ALERT_2_C Low       Ueq as Compared to Neighbors for .....        Ge3 Check
PLAT242_ALERT_2_C Low       Ueq as Compared to Neighbors for .....        Ge4 Check
PLAT242_ALERT_2_C Low       Ueq as Compared to Neighbors for .....        Ge6 Check
PLAT242_ALERT_2_C Low       Ueq as Compared to Neighbors for .....        Ge7 Check
PLAT242_ALERT_2_C Low       Ueq as Compared to Neighbors for .....        Ge8 Check
PLAT242_ALERT_2_C Low       Ueq as Compared to Neighbors for .....        Si2 Check
PLAT242_ALERT_2_C Low       Ueq as Compared to Neighbors for .....        Si3 Check
PLAT242_ALERT_2_C Low       Ueq as Compared to Neighbors for .....        Si4 Check
PLAT242_ALERT_2_C Low       Ueq as Compared to Neighbors for .....        Si5 Check
PLAT242_ALERT_2_C Low       Ueq as Compared to Neighbors for .....        Si6 Check
```

Alert level G

```
PLAT005_ALERT_5_G No _iucr_refine_instructions_details  in the CIF     Please Do !
```

---

  0 **ALERT level A** = Most likely a serious problem - resolve or explain
  1 **ALERT level B** = A potentially serious problem, consider carefully
 16 **ALERT level C** = Check. Ensure it is not caused by an omission or oversight
  1 **ALERT level G** = General information/check it is not something unexpected

```
  0 ALERT type 1 CIF construction/syntax error, inconsistent or missing data
 14 ALERT type 2 Indicator that the structure model may be wrong or deficient
  2 ALERT type 3 Indicator that the structure quality may be low
  1 ALERT type 4 Improvement, methodology, query or suggestion
  1 ALERT type 5 Informative message, check
```

Datablock: 24_728j

Bond precision: Si- C = 0.0059 A   Wavelength=0.71073

Cell:	a=9.755(2)	b=9.882(2)	c=33.400(7)
	alpha=88.55(3)	beta=83.27(3)	gamma=66.97(3)
Temperature:	293 K		

	Calculated	Reported
Volume	2942.0(13)	2941.9(10)
Space group	P -1	P-1
Hall group	-P 1	?
Moiety formula	C20 H60 Ge2 Si8	?
Sum formula	C20 H60 Ge2 Si8	C30 H90 Ge3 Si12
Mr	670.62	1005.87
Dx,g cm-3	1.136	1.136
Z	3	2
Mu (mm-1)	1.785	1.785
F000	1068.0	1068.0
F000'	1070.91	
h,k,lmax	12,12,41	12,12,41
Nref	12028	11865
Tmin,Tmax	0.646,0.807	0.664,0.982
Tmin'	0.634	

Correction method= MULTI-SCAN

Data completeness= 0.986 Theta(max)= 26.360

R(reflections)= 0.0582(7705) wR2(reflections)= 0.1358(11865)

S = 0.979 Npar= Npar = 543

The following ALERTS were generated. Each ALERT has the format
test-name_ALERT_alert-type_alert-level.
Click on the hyperlinks for more details of the test.

Alert level C

```
PLAT165_ALERT_3_C Nr. of Status R Flagged Non-Hydrogen Atoms .....          2
PLAT234_ALERT_4_C Large Hirshfeld Difference Si16   --  C33     ..       0.19 Ang.
PLAT241_ALERT_2_C High      Ueq as Compared to Neighbors for .....        C25 Check
PLAT242_ALERT_2_C Low       Ueq as Compared to Neighbors for .....        Si5 Check
PLAT242_ALERT_2_C Low       Ueq as Compared to Neighbors for .....        Si6 Check
PLAT242_ALERT_2_C Low       Ueq as Compared to Neighbors for .....        Si7 Check
PLAT242_ALERT_2_C Low       Ueq as Compared to Neighbors for .....        Si8 Check
```

Alert level G

```
PLAT005_ALERT_5_G No _iucr_refine_instructions_details  in the CIF     Please Do !
PLAT045_ALERT_1_G Calculated and Reported Z Differ by ............       1.50 Ratio
PLAT152_ALERT_1_G The Supplied and Calc. Volume s.u. Differ by ...          3 Units
PLAT154_ALERT_1_G The su's on the Cell Angles are Equal ..........    0.03000 Degree
PLAT199_ALERT_1_G Reported _cell_measurement_temperature ..... (K)        293 Check
PLAT200_ALERT_1_G Reported   _diffrn_ambient_temperature ..... (K)        293 Check
PLAT301_ALERT_3_G Main Residue  Disorder ............ Percentage =         43 Note
PLAT779_ALERT_4_G Suspect or Irrelevant (Bond) Angle in CIF .... #         57 Check
              SI15 -GE3  -SI12    1.555   1.555   1.555          19.21 Deg.
PLAT779_ALERT_4_G Suspect or Irrelevant (Bond) Angle in CIF .... #         62 Check
              SI11 -GE3  -SI16    1.555   1.555   1.555          22.81 Deg.
PLAT779_ALERT_4_G Suspect or Irrelevant (Bond) Angle in CIF .... #         64 Check
```

```
             SI13 -GE3  -SI9     1.555   1.555   1.555             26.45 Deg.
PLAT779_ALERT_4_G Suspect or Irrelevant (Bond) Angle in CIF .... #         71 Check
             SI10 -GE3  -SI14    1.555   1.555   1.555             25.05 Deg.
PLAT779_ALERT_4_G Suspect or Irrelevant (Bond) Angle in CIF .... #        137 Check
             SI14 -C25  -SI10    1.555   1.555   1.555             31.88 Deg.
PLAT811_ALERT_5_G No ADDSYM Analysis: Too Many Excluded Atoms ....          ! Info
```

```
   0 ALERT level A = Most likely a serious problem - resolve or explain
   0 ALERT level B = A potentially serious problem, consider carefully
   7 ALERT level C = Check. Ensure it is not caused by an omission or oversight
  13 ALERT level G = General information/check it is not something unexpected

   5 ALERT type 1 CIF construction/syntax error, inconsistent or missing data
   5 ALERT type 2 Indicator that the structure model may be wrong or deficient
   2 ALERT type 3 Indicator that the structure quality may be low
   6 ALERT type 4 Improvement, methodology, query or suggestion
   2 ALERT type 5 Informative message, check
```

Datablock: 25_696j

```
Bond precision:  Si- C = 0.0227 A        Wavelength=0.71073

Cell:            a=9.2278(18)     b=32.901(7)        c=12.868(5)
                 alpha=90         beta=117.97(2)     gamma=90
Temperature:     150 K

                Calculated                 Reported
Volume          3450.4(18)                 3450.4(17)
Space group     P 21/c                     P2(1)/c
Hall group      -P 2ybc                    ?
Moiety formula  C18 H54 Ge2 Si7            ?
Sum formula     C18 H54 Ge2 Si7            C18 H54 Ge2 Si7
Mr              612.46                     612.42
Dx,g cm-3       1.179                      1.179
Z               4                          4
Mu (mm-1)       1.991                      1.991
F000            1296.0                     1296.0
F000'           1299.55
h,k,lmax        10,39,15                   10,39,15
Nref            6054                       6052
Tmin,Tmax       0.787,0.819                0.587,0.826
Tmin'           0.550

Correction method= MULTI-SCAN

Data completeness= 1.000          Theta(max)= 25.000

R(reflections)= 0.1158( 5107)     wR2(reflections)= 0.2435( 6052)
```

S = 1.217 Npar= Npar = 286

The following ALERTS were generated. Each ALERT has the format
test-name_ALERT_alert-type_alert-level.
Click on the hyperlinks for more details of the test.

Alert level A

```
PLAT213_ALERT_2_A Atom C14             has ADP max/min Ratio .....        5.4 prolat
PLAT213_ALERT_2_A Atom C15             has ADP max/min Ratio .....        5.3 prolat
```

Alert level B

```
PLAT213_ALERT_2_B Atom C7              has ADP max/min Ratio .....        4.9 prolat
PLAT230_ALERT_2_B Hirshfeld Test Diff for    Si2    --  C4      ..       12.5 su
PLAT230_ALERT_2_B Hirshfeld Test Diff for    Si4    --  C10     ..       11.8 su
PLAT230_ALERT_2_B Hirshfeld Test Diff for    Si5    --  C12     ..       17.2 su
PLAT234_ALERT_4_B Large Hirshfeld Difference Si3    --  C7      ..       0.26 Ang.
PLAT241_ALERT_2_B High      Ueq as Compared to Neighbors for .....        C16 Check
PLAT242_ALERT_2_B Low       Ueq as Compared to Neighbors for .....        Si2 Check
PLAT242_ALERT_2_B Low       Ueq as Compared to Neighbors for .....        Si5 Check
```

Alert level C

```
RFACG01_ALERT_3_C  The value of the R factor is > 0.10
            R factor given   0.116
RINTA01_ALERT_3_C  The value of Rint is greater than 0.12
            Rint given   0.133
PLAT020_ALERT_3_C The value of Rint is greater than 0.12 .........      0.133
PLAT082_ALERT_2_C High R1 Value ..................................       0.12 Why ?
PLAT213_ALERT_2_C Atom Si3             has ADP max/min Ratio .....        3.2 prolat
PLAT213_ALERT_2_C Atom C6              has ADP max/min Ratio .....        3.5 prolat
PLAT213_ALERT_2_C Atom C13             has ADP max/min Ratio .....        3.1 prolat
PLAT213_ALERT_2_C Atom C16             has ADP max/min Ratio .....        3.4 prolat
PLAT220_ALERT_2_C Large Non-Solvent  C     Ueq(max)/Ueq(min) Range        5.2 Ratio
PLAT220_ALERT_2_C Large Non-Solvent Si     Ueq(max)/Ueq(min) Range        4.0 Ratio
PLAT222_ALERT_3_C Large Non-Solvent    H    Uiso(max)/Uiso(min) ..        5.2 Ratio
PLAT234_ALERT_4_C Large Hirshfeld Difference Si3    --  C8      ..       0.22 Ang.
PLAT234_ALERT_4_C Large Hirshfeld Difference Si4    --  C9      ..       0.22 Ang.
PLAT242_ALERT_2_C Low       Ueq as Compared to Neighbors for .....        Ge1 Check
PLAT242_ALERT_2_C Low       Ueq as Compared to Neighbors for .....        Ge2 Check
PLAT242_ALERT_2_C Low       Ueq as Compared to Neighbors for .....        Si3 Check
PLAT242_ALERT_2_C Low       Ueq as Compared to Neighbors for .....        Si4 Check
PLAT250_ALERT_2_C Large U3/U1 Ratio for Average U(i,j) Tensor ....        2.5 Note
PLAT413_ALERT_2_C Short Inter XH3 .. XHn     H13B   ..  H15A    ..       2.14 Ang.
```

Alert level G

```
PLAT003_ALERT_2_G Number of Uiso or Uij Restrained non-H Atoms ...          6 Why ?
PLAT005_ALERT_5_G No _iucr_refine_instructions_details  in the CIF     Please Do !
PLAT083_ALERT_2_G SHELXL Second Parameter in WGHT Unusually Large.      39.42 Why ?
PLAT093_ALERT_1_G No su's on H-positions, refinement reported as .      mixed
PLAT301_ALERT_3_G Main Residue  Disorder ........... Percentage =          19 Note
PLAT779_ALERT_4_G Suspect or Irrelevant (Bond) Angle in CIF .... #         31 Check
              SI8  -GE1  -SI7     1.555   1.555   1.555          19.66 Deg.
PLAT779_ALERT_4_G Suspect or Irrelevant (Bond) Angle in CIF .... #         41 Check
              SI6  -GE2  -SI9     1.555   1.555   1.555          22.32 Deg.
PLAT779_ALERT_4_G Suspect or Irrelevant (Bond) Angle in CIF .... #         75 Check
              SI6  -C16  -SI9     1.555   1.555   1.555          30.00 Deg.
PLAT811_ALERT_5_G No ADDSYM Analysis: Too Many Excluded Atoms ....          ! Info
```

PLAT860_ALERT_3_G Number of Least-Squares Restraints .... 24 Note

---

2 **ALERT level A** = Most likely a serious problem - resolve or explain
8 **ALERT level B** = A potentially serious problem, consider carefully
19 **ALERT level C** = Check. Ensure it is not caused by an omission or oversight
10 **ALERT level G** = General information/check it is not something unexpected

1 ALERT type 1 CIF construction/syntax error, inconsistent or missing data
24 ALERT type 2 Indicator that the structure model may be wrong or deficient
6 ALERT type 3 Indicator that the structure quality may be low
6 ALERT type 4 Improvement, methodology, query or suggestion
2 ALERT type 5 Informative message, check

Datablock: 26_945j

Bond precision: C-C = 0.0035 A Wavelength=0.71073

Cell:	a=14.513(2)	b=14.513(2)	c=10.772(2)
	alpha=90	beta=90	gamma=120
Temperature:	100 K		

	Calculated	Reported
Volume	1964.9(8)	1965.0(6)
Space group	R 3	R3
Hall group	R 3	?
Moiety formula	C18 H48 Ge Si4	?
Sum formula	C18 H48 Ge Si4	C18 H48 Ge Si4
Mr	449.53	449.51
Dx,g cm-3	1.140	1.140
Z	3	3
Mu (mm-1)	1.352	1.352
F000	732.0	732.0
F000’	733.50	
h,k,lmax	18,18,13	18,18,13
Nref	1788[894]	1760
Tmin,Tmax	0.707,0.743	0.672,0.755
Tmin’	0.642	

Correction method= MULTI-SCAN

Data completeness= 1.97/0.98 Theta(max)= 26.290

R(reflections)= 0.0228(1719) wR2(reflections)= 0.0542(1760)

S = 1.026 Npar= Npar = 79

The following ALERTS were generated. Each ALERT has the format
test-name_ALERT_alert-type_alert-level.
Click on the hyperlinks for more details of the test.

Alert level B

PLAT230_ALERT_2_B Hirshfeld Test Diff for C4 -- C5 .. 7.3 su

Alert level C

PLAT166_ALERT_4_C S.U's Given on Coordinates for calc-flagged H4

Alert level G

PLAT005_ALERT_5_G No _iucr_refine_instructions_details in the CIF Please Do !
PLAT033_ALERT_4_G Flack x Value Deviates > 2*sigma from Zero 0.040
PLAT104_ALERT_1_G The Reported Crystal System is Inconsistent with R3 Check
PLAT152_ALERT_1_G The Supplied and Calc. Volume s.u. Differ by ... 2 Units
PLAT164_ALERT_4_G Nr. of Refined C-H H-Atoms in Heavy-Atom Struct. 1 Note

---

0 **ALERT level A** = Most likely a serious problem - resolve or explain
1 **ALERT level B** = A potentially serious problem, consider carefully
1 **ALERT level C** = Check. Ensure it is not caused by an omission or oversight
5 **ALERT level G** = General information/check it is not something unexpected

2 ALERT type 1 CIF construction/syntax error, inconsistent or missing data
1 ALERT type 2 Indicator that the structure model may be wrong or deficient
0 ALERT type 3 Indicator that the structure quality may be low
3 ALERT type 4 Improvement, methodology, query or suggestion
1 ALERT type 5 Informative message, check

Datablock: 28_782j

Bond precision: C-C = 0.0055 A Wavelength=0.71073

Cell:	a=14.643(2)	b=14.643(2)	c=10.892(2)
	alpha=90	beta=90	gamma=120
Temperature:	100 K		

	Calculated	Reported
Volume	2022.6(8)	2022.5(6)
Space group	R 3	R3
Hall group	R 3	?
Moiety formula	C18 H48 Ge5	?
Sum formula	C18 H48 Ge5	C18 H48 Ge5
Mr	627.61	627.51
Dx,g cm-3	1.546	1.546
Z	3	3
Mu (mm-1)	5.508	5.509
F000	948.0	948.0
F000’	950.50	
h,k,lmax	18,18,13	18,18,13
Nref	1838[919]	1789
Tmin,Tmax	0.171,0.414	0.249,0.473
Tmin’	0.133	

Correction method= MULTI-SCAN

Data completeness= 1.95/0.97   Theta(max)= 26.290

R(reflections)= 0.0289(1765)   wR2(reflections)= 0.0705(1789)

S = 1.026   Npar= Npar = 76

The following ALERTS were generated. Each ALERT has the format
test-name_ALERT_alert-type_alert-level.
Click on the hyperlinks for more details of the test.

Alert level G

PLAT005_ALERT_5_G No _iucr_refine_instructions_details in the CIF Please Do !
PLAT104_ALERT_1_G The Reported Crystal System is Inconsistent with R3 Check
PLAT152_ALERT_1_G The Supplied and Calc. Volume s.u. Differ by ... 2 Units

---

0 **ALERT level A** = Most likely a serious problem - resolve or explain
0 **ALERT level B** = A potentially serious problem, consider carefully
0 **ALERT level C** = Check. Ensure it is not caused by an omission or oversight
3 **ALERT level G** = General information/check it is not something unexpected

2 ALERT type 1 CIF construction/syntax error, inconsistent or missing data
0 ALERT type 2 Indicator that the structure model may be wrong or deficient
0 ALERT type 3 Indicator that the structure quality may be low
0 ALERT type 4 Improvement, methodology, query or suggestion
1 ALERT type 5 Informative message, check

It is advisable to attempt to resolve as many as possible of the alerts in all categories. Often the minor alerts point to easily fixed oversights, errors and omissions in your CIF or refinement strategy, so attention to these fine details can be worthwhile. In order to resolve some of the more serious problems it may be necessary to carry out additional measurements or structure refinements. However, the purpose of your study may justify the reported deviations and the more serious of these should normally be commented upon in the discussion or experimental section of a paper or in the "special_details" fields of the CIF. checkCIF was carefully designed to identify outliers and unusual parameters, but every test has its limitations and alerts that are not important in a particular case may appear. Conversely, the absence of alerts does not guarantee there are no aspects of the results needing attention. It is up to the individual to critically assess their own results and, if necessary, seek expert advice.

Publication of your CIF in IUCr journals

A basic structural check has been run on your CIF. These basic checks will be run on all CIFs submitted for publication in IUCr journals (*Acta Crystallographica*, *Journal of Applied Crystallography*, *Journal of Synchrotron Radiation*); however, if you intend to submit to *Acta Crystallographica Section C* or *E*, you should make sure that full publication checks are run on the final version of your CIF prior to submission.

Publication of your CIF in other journals

Please refer to the *Notes for Authors* of the relevant journal for any special instructions relating to CIF submission.

Validation response form

Please find below a validation response form (VRF) that can be filled in and pasted into your CIF.

```
# start Validation Reply Form
_vrf_PLAT213_25_696j
;
PROBLEM: Atom C14             has ADP max/min Ratio .....        5.4 prolat
RESPONSE: ...
;
# end Validation Reply Form
```

PLATON version of 05/02/2014; check.def file version of 05/02/2014

Datablock 9_1056j - ellipsoid plot

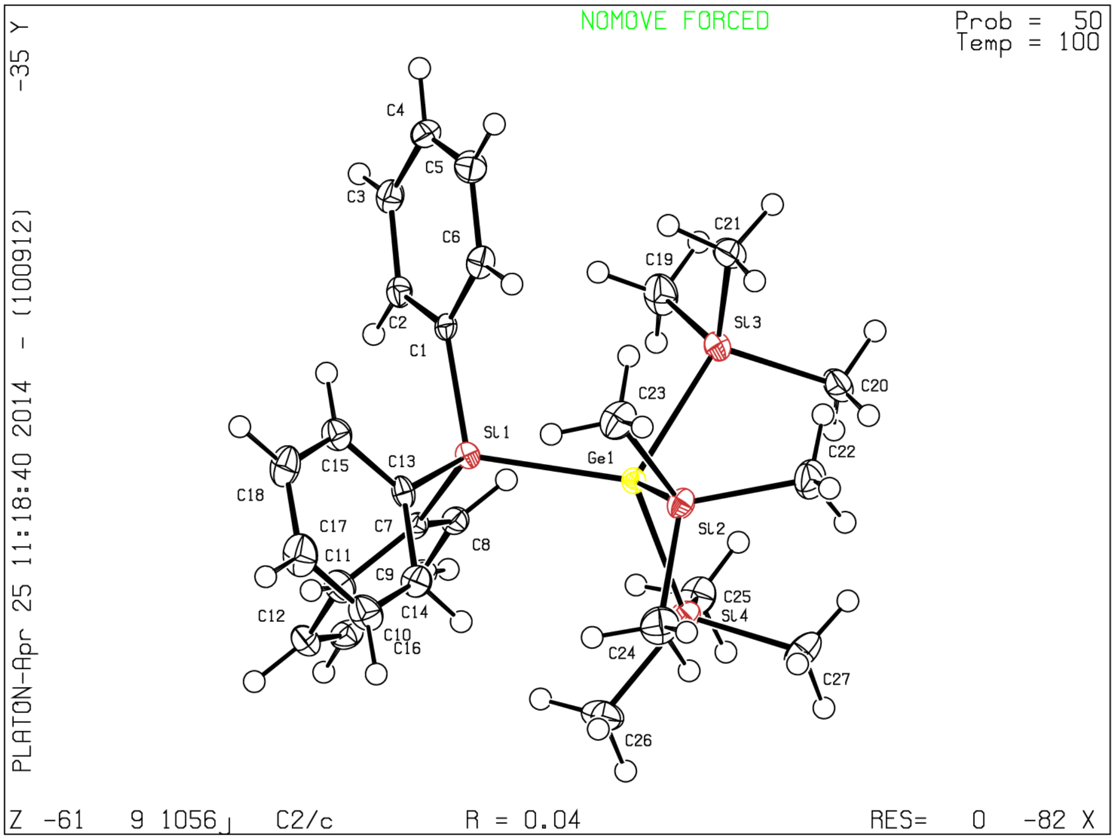

Datablock 10_845j - ellipsoid plot

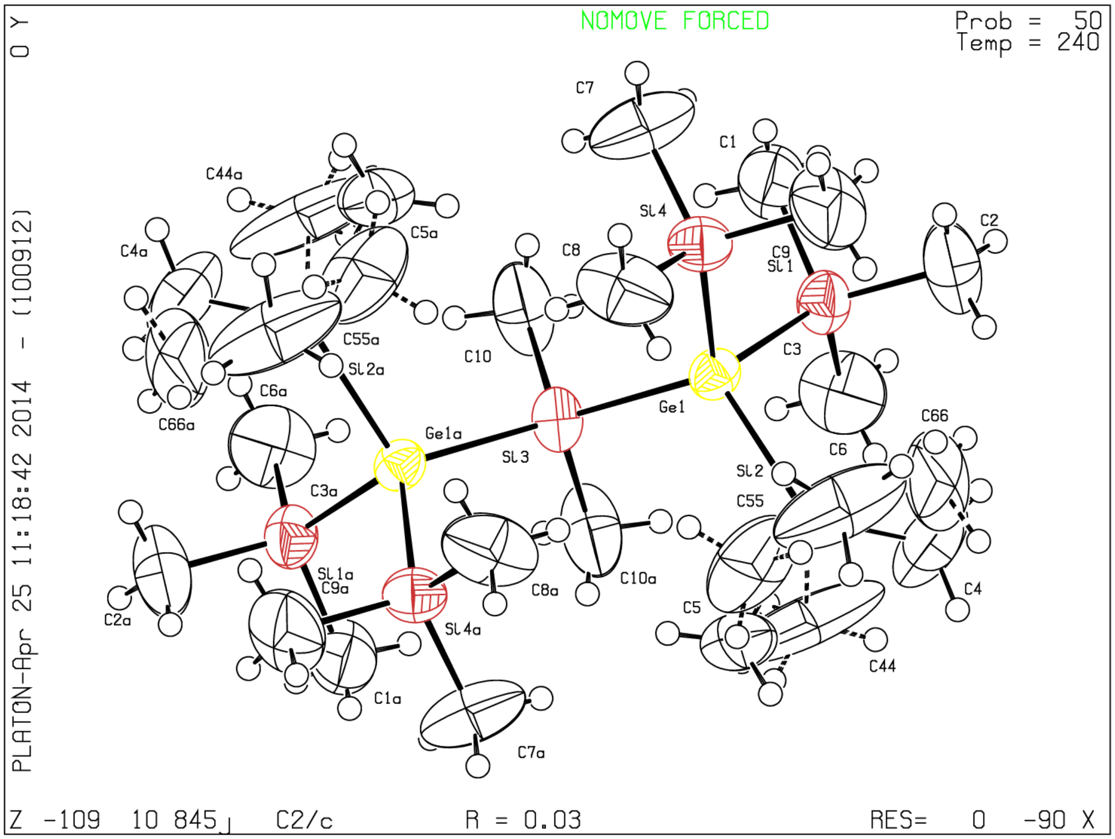

Datablock 12_649j - ellipsoid plot

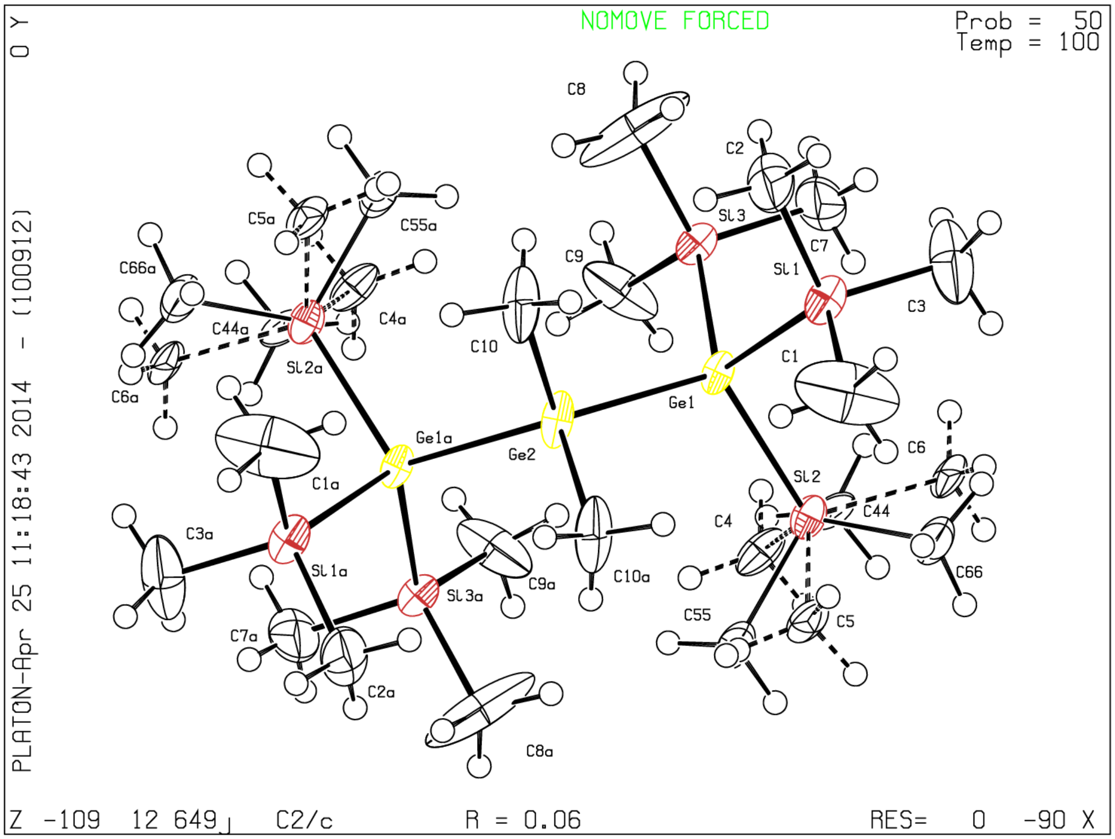

Datablock 13_729j - ellipsoid plot

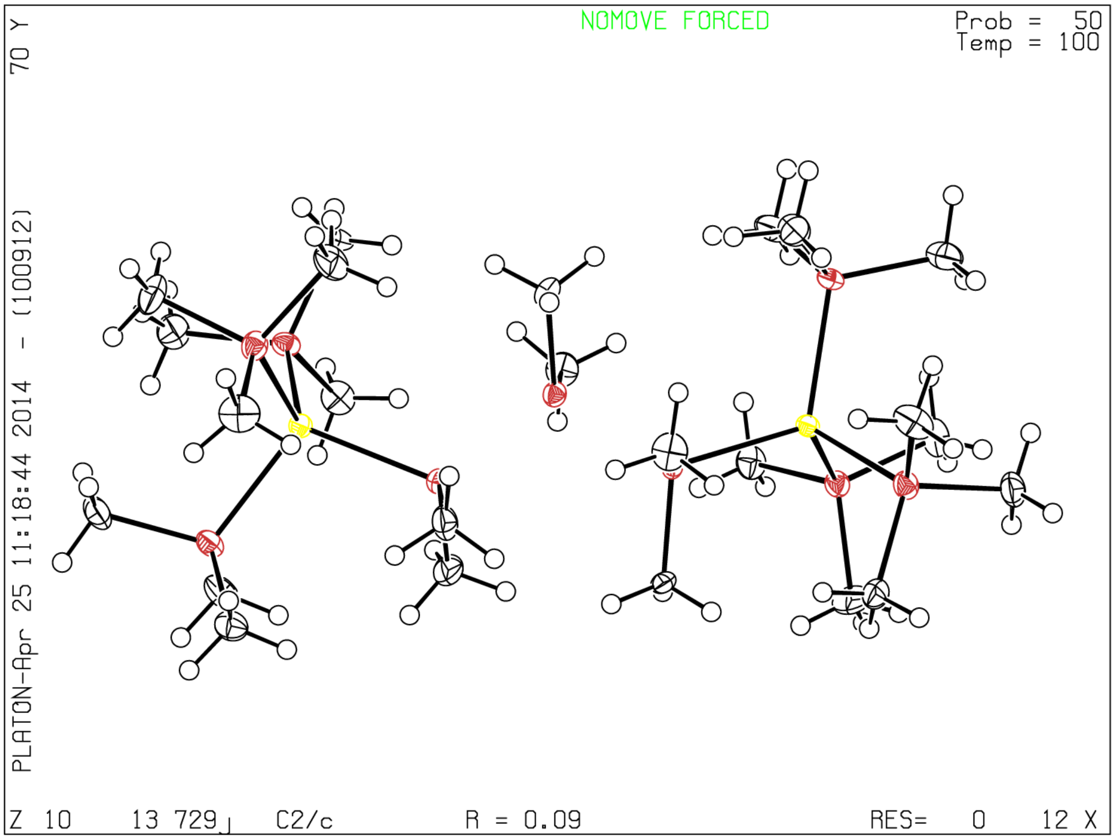

Datablock 14_760j - ellipsoid plot

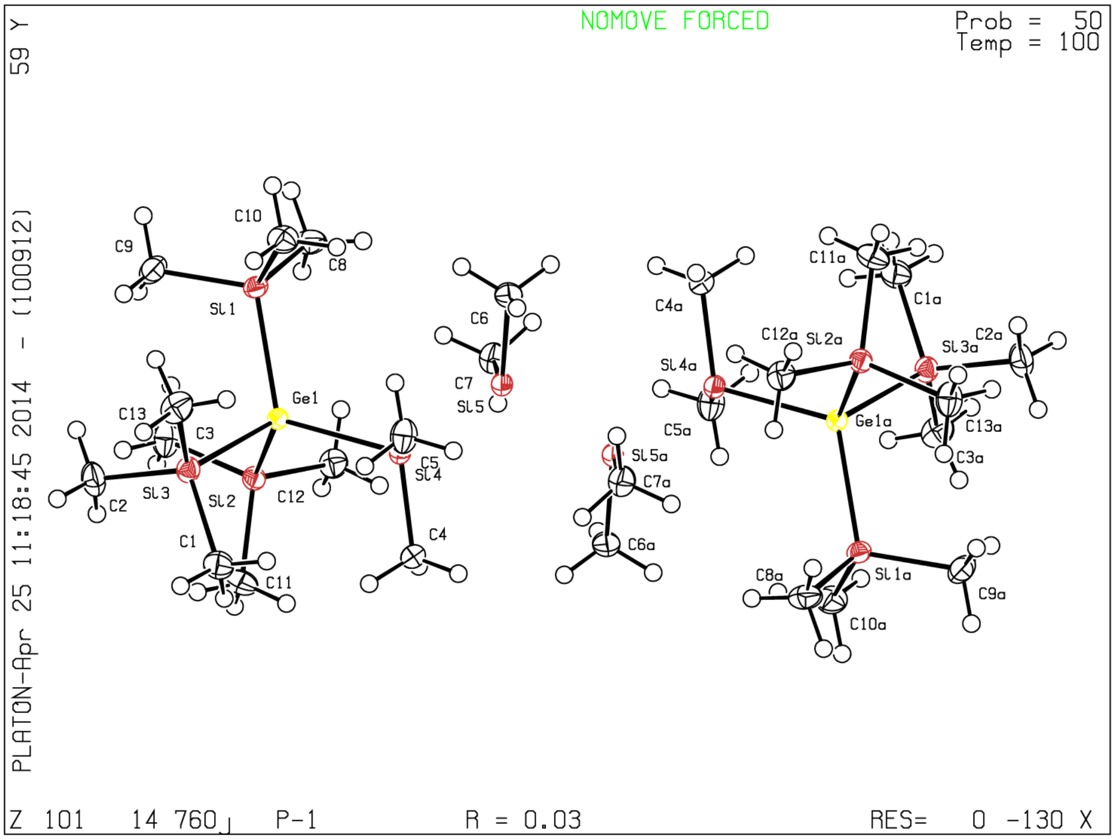

Datablock 19_661j - ellipsoid plot

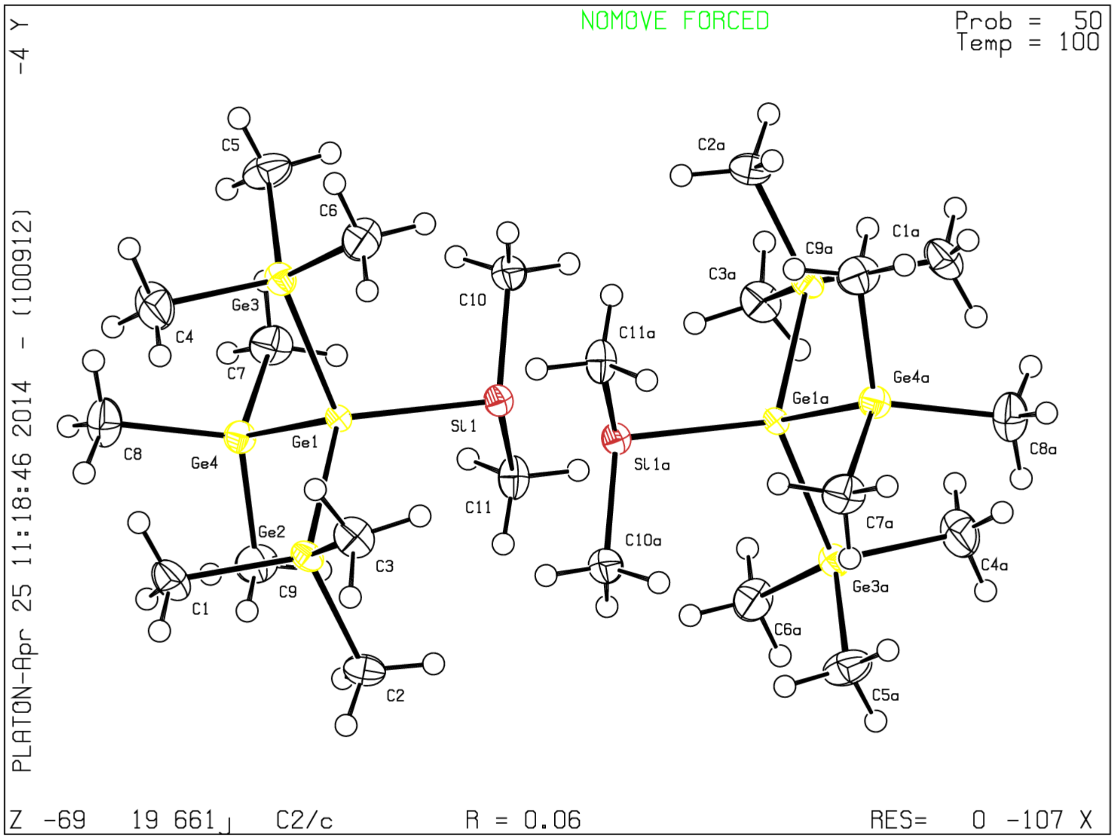

Datablock 21_660j - ellipsoid plot

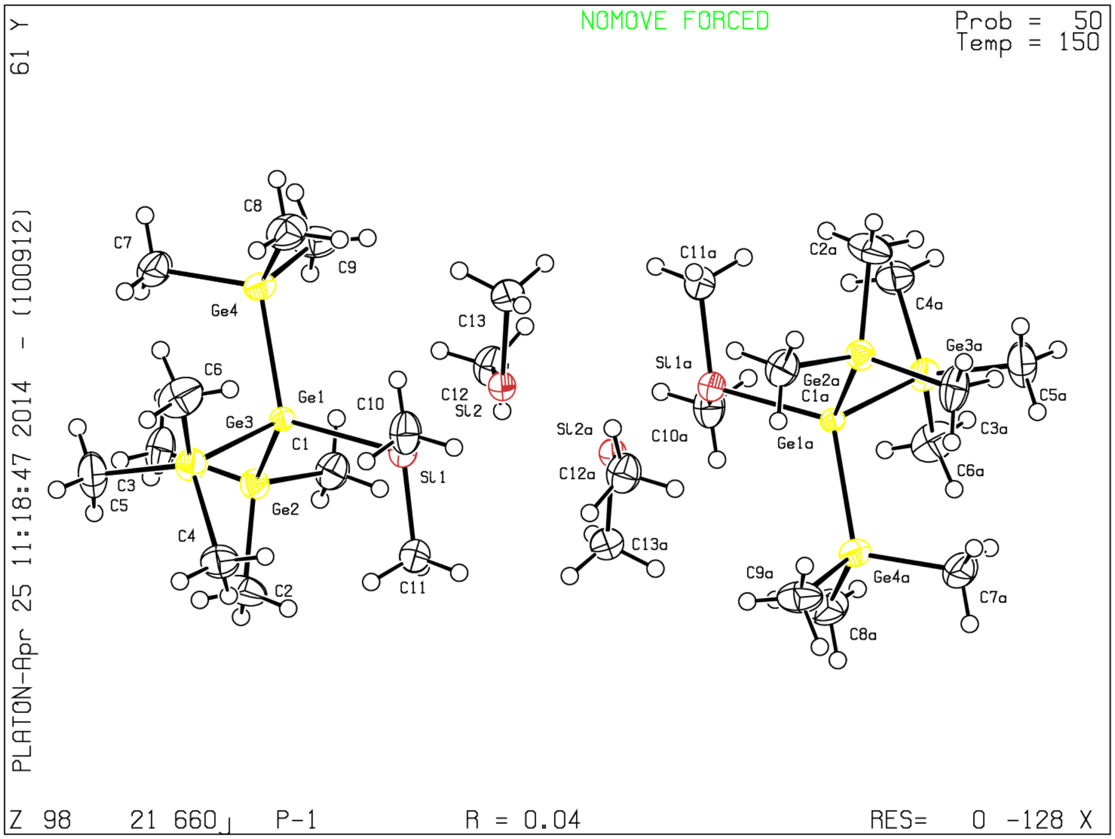

Datablock 22_672j - ellipsoid plot

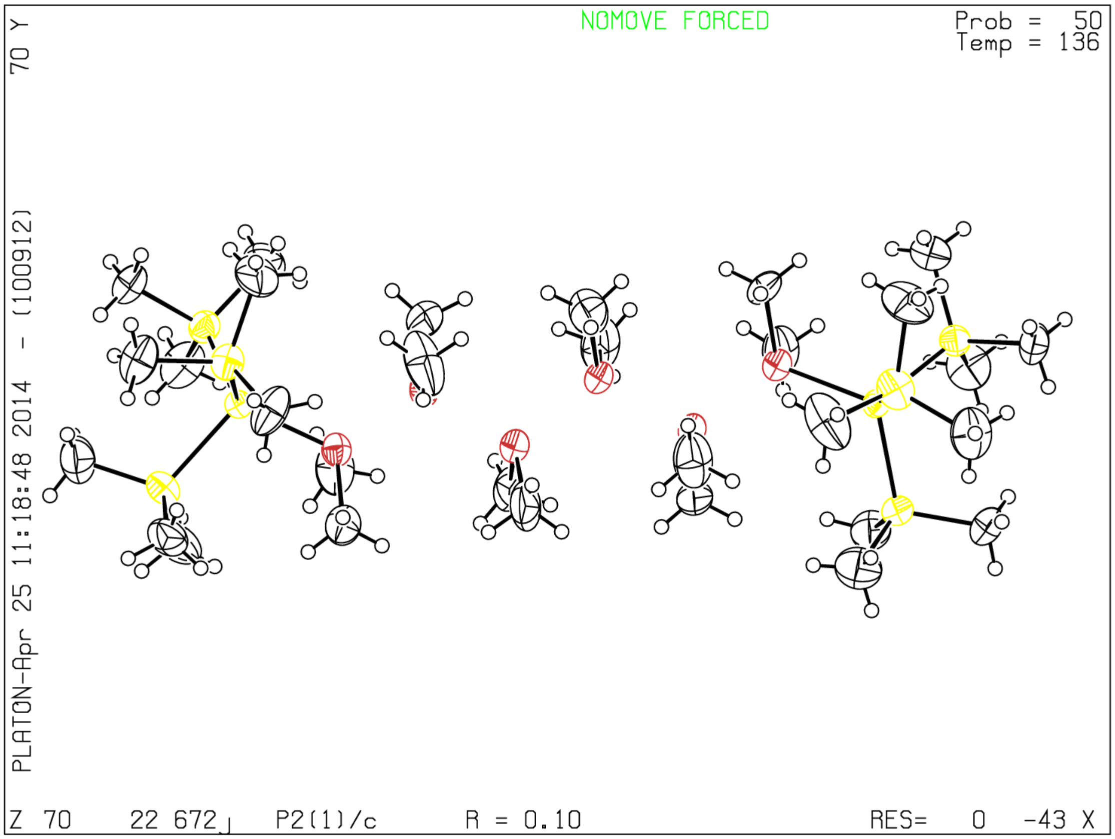

Datablock 24_728j - ellipsoid plot

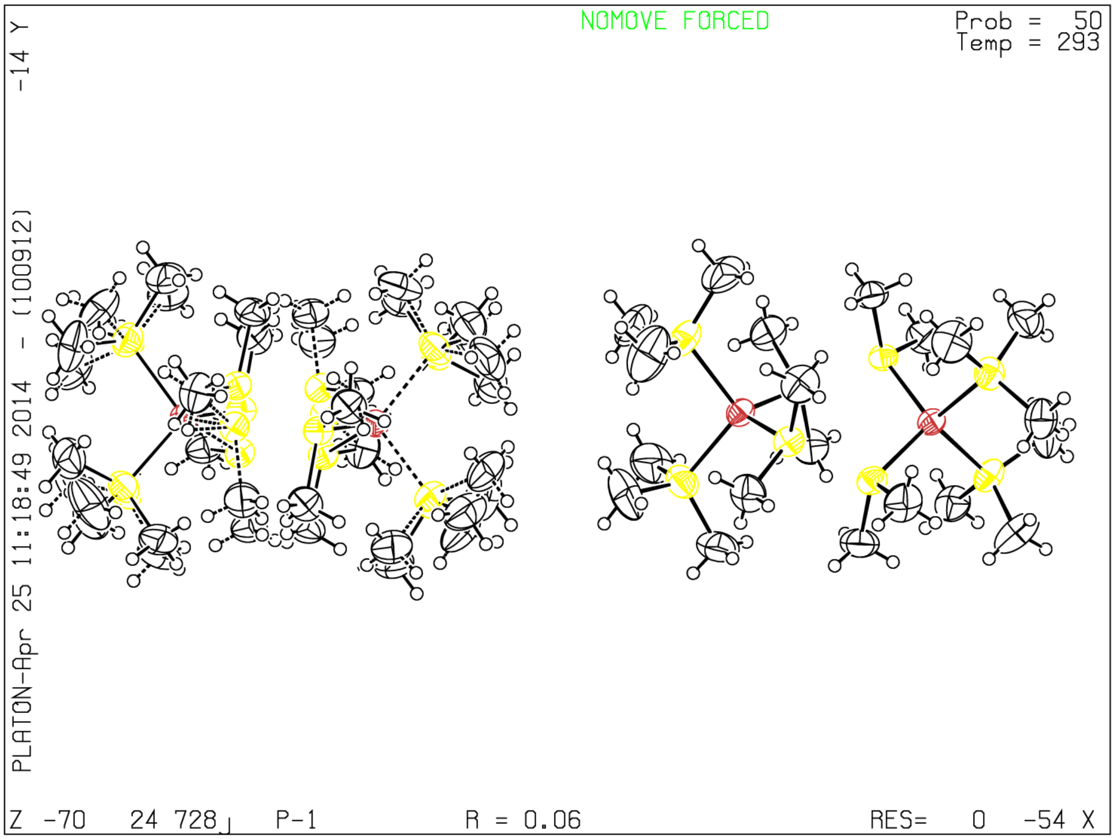

Datablock 25_696j - ellipsoid plot

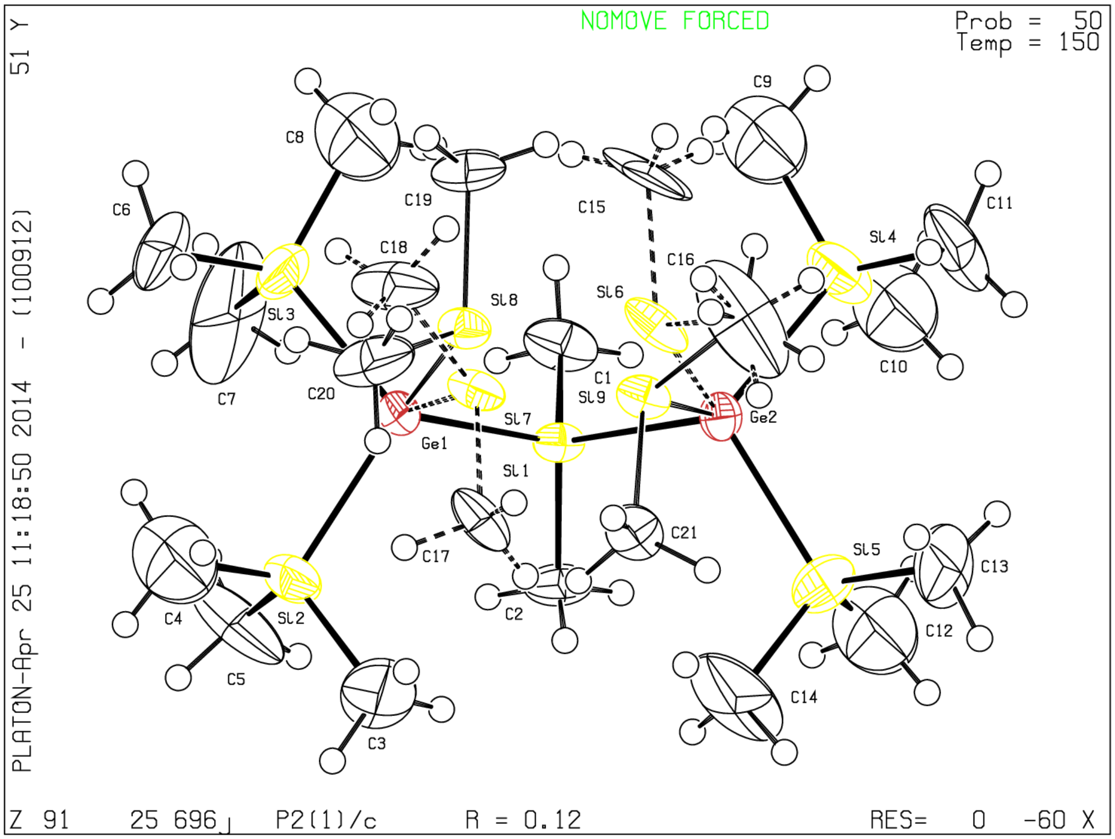

Datablock 26_945j - ellipsoid plot

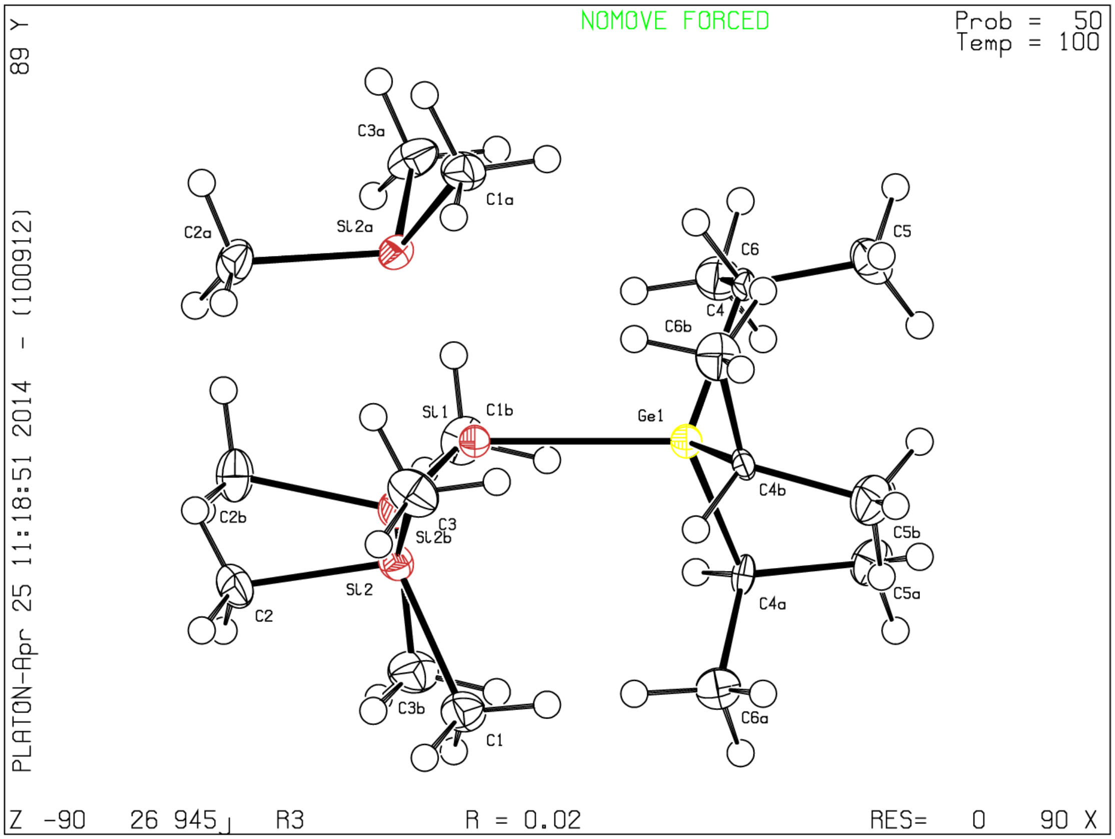

Datablock 28_782j - ellipsoid plot

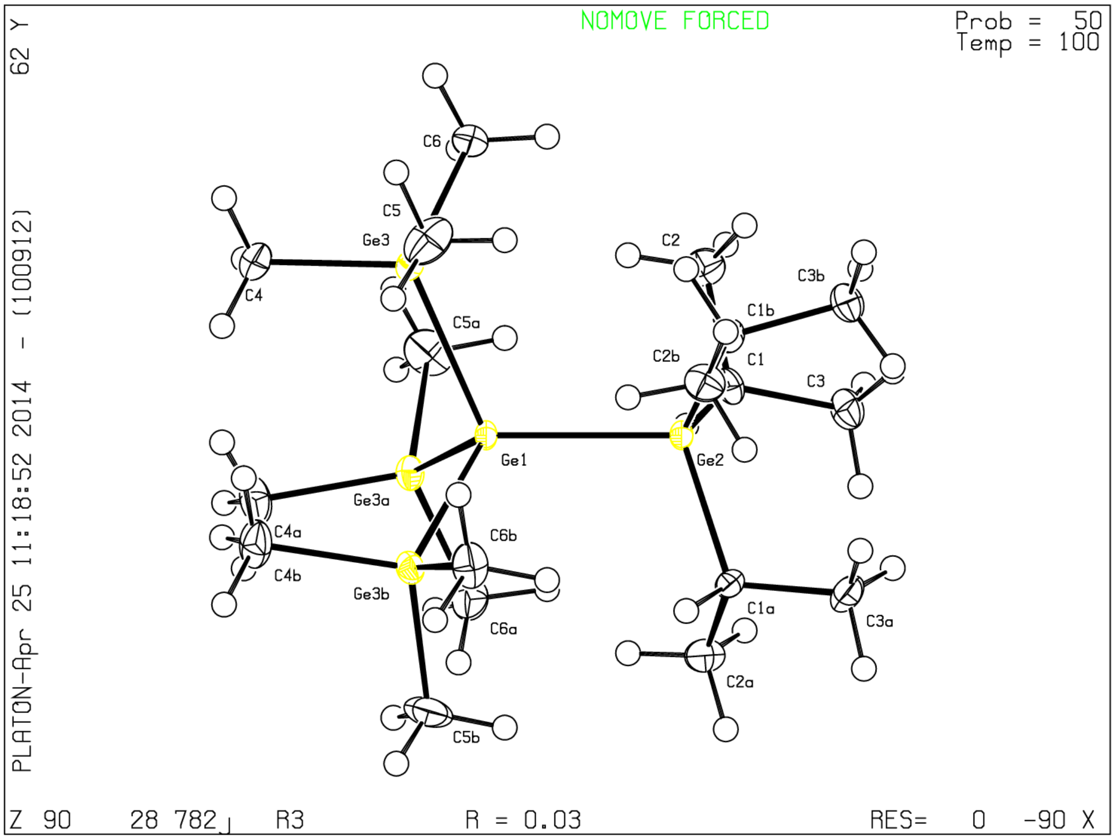

Supplement: Supplementary data 1 [file mmc1.zip › checkcif_all.pdf]
